# Community-Based Health Education Led by Women’s Groups Significantly Improved Maternal Health Service Utilization in Southern Ethiopia: A Cluster Randomized Controlled Trial

**DOI:** 10.3390/healthcare12101045

**Published:** 2024-05-18

**Authors:** Amanuel Yoseph, Wondwosen Teklesilasie, Francisco Guillen-Grima, Ayalew Astatkie

**Affiliations:** 1 School of Public Health, College of Medicine and Health Sciences, Hawassa University, Hawassa P.O. Box 1560, Ethiopia; wondeti@yahoo.com (W.T.); ayalewastatkie@gmail.com (A.A.); 2 Department of Health Sciences, Public University of Navarra, 31008 Pamplona, Spain; 3 Healthcare Research Institute of Navarra (IdiSNA), 31008 Pamplona, Spain; 4 Department of Preventive Medicine, Clinica Universidad de Navarra, 31008 Pamplona, Spain; 5 CIBER in Epidemiology and Public Health (CIBERESP), Institute of Health Carlos III, 46980 Madrid, Spain

**Keywords:** small women group, health education, antenatal care, health facility delivery, postnatal care, women, cluster randomized controlled trial, Bonferroni correction, Ethiopia

## Abstract

Objective: This study aimed to evaluate the effect of health education intervention (HEI) on maternal health service utilization (MHSU) in southern Ethiopia. Methods: From 10 January to 1 August 2023, a community-based, two-arm, parallel-group cluster randomized controlled trial (cRCT) was conducted among pregnant mothers in the Northern Zone of Sidama National Regional State, Ethiopia. We utilized multilevel mixed-effects modified Poisson regression with robust variance to control for the effects of clustering and potential confounders. The level of significance was adjusted for multiple comparisons. Results: The overall utilization of at least one antenatal care (ANC) visit was 90.2% in the treatment group and 59.5% in the comparator group (χ^2^ = 89.22, *p* < 0.001). Health facility delivery (HFD) utilization was considerably different between the treatment group (74.3%) and the comparator group (50.8%) (χ^2^ = 70.50, *p* < 0.001). HEI significantly increased ANC utilization (adjusted risk ratio [ARR]: 1.32; 99% CI: 1.12–1.56) and HFD utilization (ARR: 1.24; 99% CI: 1.06–1.46). The utilization of at least one postnatal care (PNC) service was 65.4% in the treatment group and 52.1% in the comparator group (χ^2^ = 19.51, *p* = 0.01). However, after controlling for the effects of confounders and clustering, the impact of HEI on PNC utilization was insignificant between the two groups (ARR: 1.15; 99% CI: 0.89–1.48). Conclusion: A community-based HEI significantly increased ANC and HFD utilization but did not increase PNC utilization. Expanding the HEI with certain modifications will have a superior effect on improving MHSU. Trial registration number: NCT05865873.

## 1. Introduction 

Maternal mortality is high worldwide, with 223 maternal deaths per 100,000 live births (LBs) in 2020 [1]. It will take an annual reduction rate of 11.6% to bring the global maternal mortality ratio (MMR) below 70 by 2030, a rate seldom achieved at a country level [1,2]. The MMR is disproportionately high in low- and middle-income nations (almost 95% of total maternal mortalities) [3]. Though the MMR reduced by over 34% worldwide between 2000 and 2022, significant efforts and commitments are required in low- and middle-income countries, notably in Sub-Saharan Africa (SSA) and Asia, to achieve “target 1” of sustainable development goal 3 [4,5]. 

Ethiopia is one of the nations in the SSA with a high maternal mortality rate [1,2,3,5]. According to the 2016 Ethiopian Demographic and Health Survey (EDHS), there were 412 maternal mortalities per 100,000 LBs [6]. Also, maternal mortality varies greatly between Ethiopia’s regional states. For example, it ranged from 74 to 548 deaths per 100,000 LBs in the Tigray regional state and Afar region [7]. In the Sidama region, the MMR was 419 per 100,000 LBs, with the Aroresa district having the highest rate of 1142 mortalities per 100,000 LBs [8].

Worldwide maternal survival has improved in the previous two decades due to several initiatives [4]. Nonetheless, many more survivors suffer from severe conditions such as an obstetric fistula and ruptured uterus, which can have long-term consequences [1,9]. Maternal mortality has far-reaching implications for families, societies, and nations, with an impact that spans generations. Complications that cause women’s impairments and mortality negatively impact their newborns and the children they care for [2,10]. 

Maternal death can be avoided by taking basic preventative steps and making enough care accessible during crucial times (pregnancy, childbirth, and postpartum) [1,2]. Furthermore, MHSU, which includes access to high-quality care, is thought to be tremendously helpful in reducing the burden of maternal illness and death, particularly in low-resource settings [1,2,4,11]. Nevertheless, MHSU can be poor in developing nations, predominantly in SSA [2], and Ethiopia is no exception [12].

According to the 2019 Mini EDHS report, 74% of women utilized ANC services; 43% of mothers had four or more ANC utilization visits during their most current pregnancy; over half (52%) of all deliveries happened at home; and merely 34% of women in Ethiopia received a PNC visit within the first two days after delivery. Also, considerable regional, rural, and urban disparities in maternal health service (MHS) utilization persist [12]. Furthermore, MHSU was poor in the Sidama region, wherein merely 45% of mothers utilized at least one ANC service, 40.7% had skilled deliveries, and 14.3% utilized PNC [13]. Several interconnected determinants have contributed to the limited MHSU, such as socioeconomic, demographic, and community determinants; health facility or organizational-related determinants; health care providers; women’s obstetric characteristics; perceived quality of health services; lack of service access; poor knowledge of obstetric danger signs (ODSs); health system functioning; dearth of decision-making authority; delay in receiving treatment; infrastructure; and socio-cultural and traditional practices [14,15,16,17,18,19,20,21,22,23]. 

Following the philosophy of primary health care, the Ethiopian government has been implementing multi-dimensional approaches, initiatives, and strategies to address universal inaccessibility of service and low MHSU. Among the measures are the formulation of an extensive 20-year health sector development agenda [24], a growth and transformation plan [25], and a national reproductive health strategy [26]. In addition, the delivery of free MHS and ambulance services for mothers, the teaching and hiring of health professionals, predominantly midwives and health extension workers (HEWs) in rural settings, the expansion of health facility building, and reorganizing community involvement utilizing the Women Development Army (WDA) have been undertaken [25]. 

The Ethiopian government has made efforts, but the country’s MHSU is still low overall and very low in rural areas [12]. Hence, health education could serve as one of the approaches to bring a sustainable positive or desired health behavior change. It is a method of developing the desired behavior change focused on education and communication. The assumption is that through education and communication with individuals, women and communities can, in one way or another, be influenced to act in ways that will make their lives healthier and safer [27,28].

The HEI is fundamental to increasing MHSU [29,30,31]. However, the effect of a HEI on MHSU has not been broadly investigated, and the prevailing evidence shows contradictory findings [29,30,32]. For instance, the quasi-experimental study conducted in Edu, Kwara State, Nigeria, reported a considerable increase in ANC utilization. However, the limitations of this study are the lack of appropriate randomization and control, the use of a purposive sampling method, and inadequate power [33]. A study conducted in South Sudan reported mixed results. The HEI significantly improved skilled birth attendance (SBA) utilization but did not increase PNC utilization [31].

On the other hand, the findings from Kwara and Sokoto States of Nigeria reported that the HEI positively affected the utilization of SBA and PNC services [29,30,32,34]. However, a study conducted in Latin America found no significant effect of the HEI on the utilization of health facility services [35]. Moreover, a survey from community antenatal clinics showed that peer-supported workers’ health education was unsuccessful in enhancing SBA utilization [36]. 

However, because of epidemiological and statistical drawbacks such as purposive sampling, lack of randomization, inadequate power, and a small sample size, the validity and reliability of the evidence provided by these studies are low. These studies were also quasi-experimental, which means they lacked specific characteristics of actual experiments, like randomly assigning study participants to treatment and comparator groups, which often resulted in confounding difficulties with establishing causality [29,30,32]. Additionally, these studies used the chi-square test to analyze the effect of the intervention on MHSU, but they did not account for the impact of clusters and confounders, so they had low internal validity, which is a prerequisite for external validity. Thus, the generalizability of their findings is low for the study area and other similar settings [37]. Moreover, few cRCTs examined the impact of community-based intervention on MHSUs in low-income nations, including Ethiopia. The best evidence regarding whether or not community-based health education intervention has the estimated causal effect on MHSU can be obtained from research using a cRCT. Therefore, given the limited comprehensive studies on the impact of HEI on MHSU, this study aimed to evaluate the effect of HEI on MHSU in southern Ethiopia. The current trial seeks to answer the following research question: does community-based health education intervention facilitated by small women’s groups significantly affect MHSU among pregnant women compared to routine health education provided at health facilities? 

## 2. Methods and Materials

### 2.1. Study Area

The study was performed in the northern zone, one of the four zones in Sidama National Regional State, Ethiopia [38]. It is approximately 273 km south of Addis Ababa, Ethiopia’s capital. According to the Sidama Regional Health Bureau’s 2022 report, the northern zone had a population of 1,290,000. The total number of reproductive-age women was 300,570, with 12,023 expected pregnancies. The zone has 162 *kebeles* (the lowermost administrative unit in the country) with 382,000 households, eight rural districts, and two town administrations. Most people reside in rural areas, where agriculture is the primary source of income. For urban residents, trade is the primary source of income. The northern zone has four primary hospitals, one general hospital, 36 health centers, and 144 health posts that are currently functional and provide MHSs. Based on the Regional Health Bureau 2022 report, the primary cause of maternal mortality is hemorrhage and obstructed labor [39]. The zone was selected considering transportation accessibility and a favorable geographic location that would allow for oversight and improve the likelihood of resolving any possible issues during the intervention’s implementation.

### 2.2. Study Design and Population 

From 10 January to 1 August 2023, a community-based, two-arm, parallel-group cRCT was conducted among pregnant mothers in the Northern Zone of Sidama National Regional State, Ethiopia. This study regarded Kebeles, lower administrative units within districts, as clusters. We included all pregnant mothers who lived in the study area for at least half a year and had a gestational age of ≤12 weeks. Pregnant women who planned to shift residences during the intervention’s implementation or had critical health problems were excluded from our study. From the perspective of this study, critical maternal health problems comprise severe mental illness, chronic diseases, and severe hyperemesis gravidarum that necessitate close hospital monitoring based on reports of women development (WDT) leaders and HEWs.

Using the established WDT leaders and HEWs, pregnancy detection protocols, and monthly menstrual checks, we found 1126 pregnancies. The WDT leaders and HEWs conducted house-to-house censuses of all eligible houses to see if pregnant women lived there. A two-stage screening approach was used to identify pregnant women. Women were first interviewed regarding pregnancy symptoms and signs. Women who mentioned symptoms and signs of pregnancy underwent additional screening, which involved a human chorionic gonadotropin (HCG) urine test. We conducted the HCG test for all women who had missed their menstrual cycle for 45 days or more. Women were enrolled in this study if the test findings were positive for pregnancy. Before randomization, HEWs and WDT leaders collected written consent from study participants after they had provided adequate information about this study. The enrollment period was open from 1 November to 31 December 2022. We reported this study based on the recommended checklist for reporting cRCTs, and the completed checklist is included as additional evidence (File S1).

### 2.3. Sample Size Computation 

Using OpenEpi version 3.01, the minimum needed sample size was determined by considering the following assumptions: Because there were no prior cRCTs on the subject, estimates of the percentage of women who utilized ANC in the comparator and treatment arms were obtained from earlier quasi-experimental research [40]. The percentage before the intervention was considered the percentage in the comparator arm, and the percentage after the intervention was considered the percentage in the treatment arm. Consequently, P1 = 23.0% (percentage of women who utilized ANC in the comparator group) and P2 = 41.4% (percentage of women who utilized ANC in the treatment group) [40], 95% confidence interval (CI), control-to-experimental group ratio 1, and 80% power. According to the above considerations, the estimated sample size for the individual-based randomization trial (IRT) was 222 for both arms (111 for the treatment arm and 111 for the comparator arm). The sample size was adjusted for the non-response rate (NRR) by dividing the adequate sample size by the anticipated response rate. The adjusted sample size for NRR was 222/0.93 = 239. 

We used a cluster randomization method to assign our study subjects into study groups because our intervention is more appropriate for delivery at the group or cluster level to minimize information contamination between arms. This design offers logistical simplicity while reducing the spillover effect of the intervention. Nevertheless, to maximize this study’s statistical power, the sample size calculation must account for the impact of clustering by computing a variance inflation factor (VIF) [41,42,43]. The number of clusters needed for this study was calculated by multiplying the interclass correlation coefficient (ICC) and the adequate sample size for both groups [41]. We accepted the ICC value of 0.05 from the range of 0.01–0.05 based on the suggestion [41,42,43] because there was no reported ICC from earlier studies. As a result, for both groups, the minimal cluster number required was 308 × 0.05 = 15.4. 

Nevertheless, 24 clusters (*kebeles*) were used in this trial to ensure the cluster’s adequacy to attain the needed power for this study to detect the intended effect [44,45]. The VIF was computed using a standard formula: [VIF = 1 + (m − 1) ICC] and, assuming an average cluster size (m) of 14 study participants from 24 clusters with equal sizes, a 0.05 ICC value [41,42,43]. The VIF of 1.65 was multiplied by the adequate sample size to adjust for the cluster effect. As a result, the minimum calculated sample size was 394 (197 in the treatment arm and 197 in the comparator arm). Similarly, P1 = 4.6% (percentage of women who utilized HFD) in the comparator group and P2 = 11.5% (percentage of women who utilized HFD) in the treatment group [40]. According to the above considerations, the estimated sample size for the IRT was 546 for both arms (273 for the treatment arm and 273 for the comparator arm). The sample size was adjusted for the non-response rate (NRR) by dividing the adequate sample size by the anticipated response rate. The adjusted sample size for the NRR was 546/0.8 = 682. This sample size was multiplied by a VIF of 1.65 to adjust for the cluster effect, and the final calculated sample size was 1126 for both arms. 

### 2.4. Randomization

After obtaining consent and enrolling each study participant, randomization was carried out. *Kebeles* were stratified according to place of residence and then assigned at random to either the treatment or the comparator group. Each district’s *kebeles* served as clusters in our study because they provided logistical ease and decreased the amount of information contamination between the two arms. Stratification decreases stratum variation and aids in balancing confounders between the two groups [44,45]. Twenty-four clusters from four randomly selected districts were included in this study. Each group was given a comparable number of clusters from each stratum to make the two arms more similar. Thus, using an SPSS random number generator, three urban *kebeles* (six *kebeles*) were assigned to each group from the four districts. Likewise, nine rural *kebeles* were assigned to each arm from the four districts (18 rural *kebeles*). Lastly, from each cluster, we recruited 47 pregnant women. 

### 2.5. Study Variables

For this study, we focused on the three MHSU variables as outcome analysis variables, namely ANC, HFD, and PNC utilization, while the previous paper examined mothers’ knowledge about ODSs and birth preparedness and complication readiness (BPCR) practice. Every outcome variable was evaluated based on the mother’s self-reports and had a binary response. Each dependent variable was coded with a ‘1’ for utilization and a ‘0’ for not utilizing the services from trained providers. Health education was the exposure or intervention variable. To reduce perinatal mortality and improve women’s care experiences, the World Health Organization (WHO) has increased its recommended number of antenatal care contacts from four to eight. Thus, we considered eight or more antenatal care utilization contacts, as per WHO recommendations on a positive pregnancy experience [46]. For six months, the treatment group was provided with standard and pre-prepared audio-based health education supplemented by posters in a limited village meeting area two times a month, while the comparator group was supplied with the standard health education program as per Ethiopian guidelines for six months [47]. We classified the covariate variables into individual and community-level covariate variables. Details of the measurement of these variables are provided in Supplementary File 1 of another publication [48]. 

### 2.6. Blinding

The intervention’s nature precluded blinding the study members or the research groups (open-label). On the other hand, the subjects’ group assignment was concealed from or unknown to the data collectors (outcome assessors).

### 2.7. The Theoretical Framework of HEI 

More research indicates that specific strategies that integrate several theories and concepts have more significant effects than others, and interventions developed with an explicit theoretical foundation or models are more successful than those without a theoretical base. The underlying mechanisms of theory-based interventions having more successful effects than interventions not guided by theories are unclear. However, it was argued that using theories well-suited to the problems and contexts investigated in the studies could explain the effectiveness of theory-based interventions [49]. Theory-based strategies may also be developed with more attention, fidelity, and structure. Thus, the most effective public health initiatives and programs are founded on comprehending health behaviors and their context [49,50]. As a result, interventions to improve health behavior are best designed when relevant behavior change theories are understood and used skillfully [49]. Research also demonstrates that interventions with the highest chance of success are founded on thoroughly comprehending the targeted health behaviors and the environmental contexts in which they occur [50]. The conceptual or theoretical framework for the intervention in the present study was based on the social cognitive theory (SCT). This theory states that a person’s likelihood of changing their health-related behavior is influenced by three main factors: self-efficacy, goals, and outcome expectations. When people believe in their abilities, they can overcome obstacles and modify their behavior [51]. SCT integrates ideas and procedures from cognitive, behavioral, and emotional behavior change models, making it easily applicable to HEI for health-seeking behavior change. A core principle of SCT is that learning occurs not only from personal experience but also from witnessing other people’s actions and the outcomes of those actions [49,51]. 

**Figure 1:**
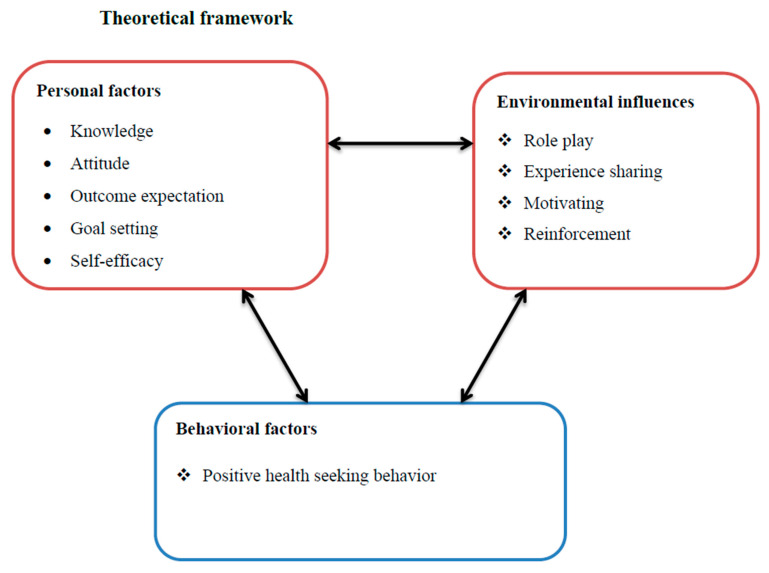
The list of constructs under each component of the SCT was adapted for this study.

Figure 1 indicates the list of constructs under each component of the SCT adapted for this study. Knowledge constructs in this intervention mainly addressed HEI titles like uncomplicated pregnancy and childbirth, ODSs and contact persons during their occurrences, a BPCR plan and its importance, and a skilled MHS and its benefits. Some selected women carried out roleplay and shared their experiences about ODS occurrence, its consequences in the community, and the benefits of skilled MHSs. In addition, WDT leaders motivated pregnant mothers and their families to utilize MHSs. The maternal outcome expectation from this intervention, which included pre-recorded HEI audio designed to educate on the complications and severity of ODSs and the benefits of MHS-uptake to overcome these complications, was further enhanced by correcting their misconceptions about MHSs. Self-efficacy-related issues include empowering pregnant mothers with the knowledge to comply with MHS-uptake (verbal persuasion) and evaluating their self-efficacy with each other for complying with MHS-uptake at the end of sessions. Goal-setting issues were addressed during their first HEI session, and pregnant mothers were informed about compliance with the ANC and PNC schedules and encouraged to set goals. Regarding the reinforcement of pregnant women, the WDT leaders reminded women about the schedule of HEI sessions and ANC appointments two days before their sessions. The environmental (social) norms linked information was obtained during group dynamics, such as sharing experiences and a brief discussion on the traditional, religious, and cultural influences of MHSU.

### 2.8. HEI Procedure

#### 2.8.1. Training of WDT Leaders and Intervention Delivery Process 

The intervention was designed to be delivered by WDT leaders who were willing to participate and were literate in *Sidaamu Afoo*, the local language. Three days of intense training on topics like uncomplicated pregnancy and childbirth, as well as knowledge of ODSs, BPCR practice, and MHSU, were provided after the recruitment. In addition, the training covered ethical issues, how to handle pre-prepared audio material and posters, and who should be contacted if they have particular concerns about the research HEI procedures. Twice a month, in a small community gathering space, WDT leaders led the HEI utilizing pre-prepared audio messages. The health education messages were designed by the principal investigator and reviewed by the research team members. A Hawassa University health education expert also reviewed the message and the tools. After several revisions, the finalized version of the health education message was prepared. A female midwife with a bachelor’s degree and media specialists received a comprehensive orientation on the high-quality audio-recorded material development method and HEI standard operating procedure. The midwife narrated the prepared document multiple times until all sounds and messages were understood clearly within the local culture and language context. Subsequently, the midwife prepared the finalized draft of the pre-prepared audio-based HEI lecture, and professionals at a Sidama Media Network or local media network studio handled the audio recording. At each health education session, the pre-recorded audio messages were played via portable Bluetooth devices called “Gepps”.

A total of 12 sessions, lasting an hour each, were held for six months to administer the HEI. A single health education presentation covered key messages regarding uncomplicated pregnancy and childbirth, knowledge of ODSs, BPCR practice, and the importance of MHSU. Encouraging women and their families to participate actively in HEI sessions was another task carried out by the WDT leaders. Each session lasted one hour, of which twenty minutes were dedicated to the pre-prepared audio-based health education lecture and forty minutes to highlighting posters, queries, and responses, which is termed the discussion period. 

Following each session, a group of women participated in a roleplay, a fundamental method of sharing stories and illustrating key points. The facilitators repeated the information to help these women internalize the main point. The women were also shown posters to reinforce the lesson or fill in any gaps from the audio lecture. During the session, any queries, ambiguities, or misinterpretations were noted and communicated to the midwife by HEWs in case they could not provide sufficient clarification. Once a month, HEWs living in specific clusters were in charge of answering inquiries. If the issues raised required a more in-depth explanation than what HEWs could offer, we recruited the midwife who narrated the audio material. After the session at a subsequent meeting, the midwife, who was not actively in the study area, explained the issues to mothers and HEWs over the phone for all women. A supervisor was hired to oversee the health education sessions in each district once a month during the study period or more repeatedly if possible challenges were encountered for quality-checking purposes. Supervisors informed issues, such as absenteeism or disagreements between WDT leaders and group members, to the principal investigator (PI). The PI discussed and resolved the problems with the WDT leaders, group members, *kebeles* leaders, and HEWs.

#### 2.8.2. Uniqueness of Current Intervention Delivery Process 

The current intervention differs in a few ways from the standard intervention [47,52]. First, the community-based aspect of the current intervention involves all pregnant mothers in *kebeles* who were arranged into groups of fifteen or fewer. WDT leaders facilitated the intervention, and the WDT leaders led a small group of pregnant women (often 15 or fewer). Thirty-eight groups of pregnant mothers were created. Therefore, the current intervention (which is decentralized) comprises pregnant mothers from *kebeles* that would typically be inaccessible. Pregnant mothers who attended health posts were given health education as part of the routine intervention, which is centered on health posts and does not consider pregnant women at home or in remote locations. Second, whereas the routine intervention is provided once a month, our HEI is provided twice a month. Regular teaching is believed to result in a greater understanding of the benefits of MHSs and increased utilization of MHSs. Third, in contrast to the standard intervention (which merely utilizes the lecture method), our HEI is clear-cut, easy to understand, rich in content, and backed by audio teaching materials that have been pre-recorded. Uniform or standard information was provided to all clusters through this audio-visual device-assisted HEI procedure to establish comparable comprehension. Fourth, this intervention identifies and enrolls pregnant women less than 12 weeks pregnant through house-to-house visits. The standard intervention provides health education to pregnant mothers probably at more than 16 weeks of gestational age. As a result, our approach is intended to increase the likelihood of women completing the continuum of care.

### 2.9. Data Collection Tools and Procedures 

We used a pre-tested, structured, face-to-face interviewer-administered questionnaire to collect data. It was taken from earlier, comparable research [14,15,16,17,18,19,20,21,22,23]. The average time taken by this study questionnaire during the face-to-face interview was 30 min, estimated at the time of the pretesting of the tool. The details of the data collection tools and procedures have been published elsewhere [48]. Data were gathered at seven weeks following the delivery or end of the PNC period. The data were collected at the women’s homes by first-degree healthcare workers and blinded to the participant intervention groups using the questionnaire (see File S2) using the Open Data Kit (ODK) application. Women’s attendance records from the WDT leaders’ reports to the PI were used to assess the women’s adherence to HEI sessions at the end of the interventions. Several measures were undertaken to reduce the possibility of bias during the intervention implementation period and the data collection period. These measures encompassed increasing the follow-up and response rates, providing extensive training to supervisors and data collectors, blinding outcome assessors to the group allocation status, and ensuring that a blinded statistician performed the randomization. WDT facilitators reminded study participants two days before the next HEI session to reduce the loss of follow-up. We gathered online data for all clusters between 28 August and 22 September 2023. The collected data were sent to the KoboToolbox server daily, and the PI monitored their completeness and quality. Immediately after data collection was completed, the PI exported the data from the server to SPSS version 26 for additional processing, cleaning, preparing, coding, categorizing, computing, and exploring before principal analysis (File S4).

### 2.10. Statistical Analysis 

We used descriptive measures of absolute frequency and percentage for categorical data presentation, whereas the mean and standard deviation (SD) for numerical data were reported after confirming the normality of the data. Using intention-to-treat analysis (ITTA), we examined the effect of the HEI on the MHSU of women initially enrolled in the trial and available during the outcome assessment period. The intervention was randomly assigned at the cluster level, but the outcome was evaluated individually. In an unadjusted analysis, the effect of the HEI on the MHSU was assessed using a chi-square test. The details of the data analysis procedure and wealth index calculation are provided elsewhere [53].

We calculated the ICC value and checked the significance of the random intercept using a mixed-effects multilevel logistic regression model. We fitted a multilevel model as per recommendation because the ICC values were greater than 5% for all outcome variables, and the random intercepts were significant [54,55]. To account for the hierarchical nature of our data [55] and provide a robust and reliable error estimate [56], we employed a multilevel modified Poisson model with robust standard error. Four models were examined. The empty model contained only the intercept; in Model 1, individual-level covariates and the intervention variable were included; in Model 2, only community-level covariates were included; and, in Model 3, the intervention variable was present along with other individual and community-level covariates. The percentage of MHSU variability attributable to the clustering variable was calculated using the ICC value. The best model for the data was identified using the log-likelihood statistic, the Bayesian information criterion (BIC), and Akaike’s information criterion (AIC). The lowest values of these characteristics or a significant likelihood ratio test can be used to identify the best-fitting model (File S3) [57]. 

Variables with *p*-values of 0.25 on bivariable analysis and other factors that demonstrate practical significance with appropriate backing from the medical literature were selected for the multivariable model [58]. We used the Bonferroni correction to adjust the significance level for the problem of multiple comparisons. The effect of the intervention was evaluated for five outcomes (two outcomes reported in another work). Thus, the adjusted significance level was calculated by dividing the pre-fixed level of significance (0.05) by the total number of outcome variables assessed for intervention effect. Accordingly, the corrected significance level was 0.05/5 = 0.01. An association was considered statistically significant when the *p*-value was less than 0.01 [59,60]. The presence and strength of a statistically significant association were evaluated using ARRs with 99% CIs. When the 99% CIs of the ARRs did not contain 1, a statistically significant association between the HEI and MHSU was declared.

## 3. Result

### 3.1. Trial Profile

**Figure 2:**
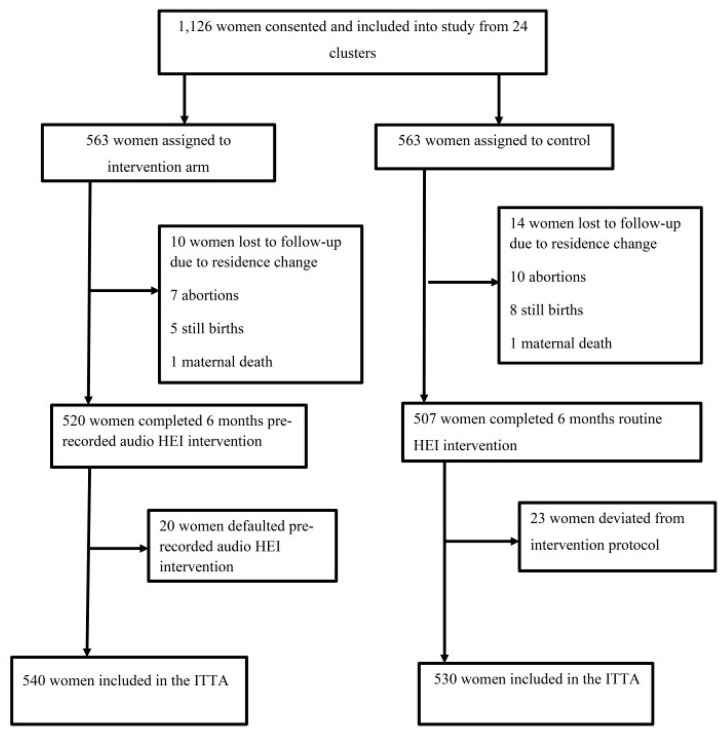
Trial profile. Note: ITTA stands for intention-to-treat analysis, and HEI stands for health education intervention.

We evaluated 1440 pregnant women during November and December 2022 to determine their inclusion in the trial based on criteria; 1126 women from 24 *kebeles* met the requirements and were enrolled for this trial. In both groups, the percentage of mothers lost to follow-up was similar (4.98% in the treatment group compared to 5.87% in the comparator group) (Figure 2). The information on the trial’s profile, such as the recruiting, eligibility, and randomization processes, was fully outlined in another paper [53].

### 3.2. Socio-Demographic Characteristics of Trial Subjects

The treatment and comparator arms were balanced in terms of most socio-demographic characteristics. The complete information on the socio-demographic and economic characteristics of the trial participants in this study has been described elsewhere [53].

### 3.3. Reproductive Health Characteristics of Trial Participants

The majority of the baseline reproductive health characteristics were comparable. The whole reproductive health characteristics of the trial participants in this study has been described elsewhere [53].

### 3.4. Description of Maternal Health Service Utilization 

The utilization of at least one ANC service was 90.6% in the treatment group and 67.0% in the comparator group, whereas eight-or-more ANC utilization was 37.8% in the treatment group and 21.9% in the comparator group (*p*-value <0.001). HFD utilization was 84.4% in the treatment group and 61.7% in the comparator group (*p*-value <0.001). Merely 21.1% of mothers in the treatment group had four or more PNC visits within six weeks after childbirth, and 15.3% in the comparator group (*p*-value = 0.01) (Table S1 of Supplemental File S3).

### 3.5. Effect of HEI on Antenatal Care Utilization 

Mothers who had obtained six months of HEI had a 32% greater likelihood of ANC utilization (ARR: 1.32; 99% CI: 1.12–1.56) than women who did not receive HEI (Table 1).

### 3.6. Effect of HEI on Eight or More Antenatal Care Utilization

The HEI has significantly improved the eight-or-more ANC utilization between the two groups (ARR = 1.51; 99% CI: 1.03–2.22) (Table 2). 

### 3.7. Effect of HEI on Health Facility Delivery Utilization

Women in the treatment group had 24% more likelihood of HFD utilization than the comparator arm (ARR = 1.24; 99% CI: 1.06–1.46) (Table 3). 

### 3.8. Effect of HEI on Postnatal Care Utilization 

After adjusting for confounders and clusters, the effect of HEI on PNC utilization was not significant between the two groups (ARR = 1.15; 99% CI: 0.89–1.48) (Table 4). 

### 3.9. Random Effect Model of Maternal Health Service Utilization 

The multilevel mixed effects modified Poisson regression with robust variance fit the data better than the standard Poisson regression model (*p* < 0.001). By using the intercept-only multilevel binary logistic model, the ICC value showed that 22.35% of the disparities in using ANC, 21.88% in using HFD, and 10.76% in using PNC could be explained by membership in *kebeles* (Table S2 of Supplementary File S3).

### 3.10. Model Selection Criteria 

The empty model was the least fit in the model fitness assessment test of ANC utilization (AIC = 2090.99, BIC = 2100.94, and log-likelihood = −1043.49). Nonetheless, there was a significant improvement in model fitness, mainly in the final model (AIC = 2083.43, BIC = 2093.38, and log-likelihood = −1019.96). As a result, the final model is the best fit compared to the other models. Similarly, in HFD and PNC, the model fitness improved considerably from the null model to the final model (Table S2 of Supplementary File S3).

## 4. Discussion

Our results show that the overall utilization of at least one ANC visit was 90.6% in the treatment group and 67.0% in the comparator group. Eight or more ANC visits were 37.8% in the treatment group and 21.9% in the comparator group. The HFD utilization was significantly higher in the treatment group (84.4%) compared to the comparator group (61.7%). HEI significantly increased ANC and HFD utilization but not PNC utilization. 

The present study showed that HEI increased ANC utilization, which is consistent with findings from the Kwara State of Nigeria [33], Balochistan of Pakistan [61], and Mirzapur of Bangladesh [62]. This finding is consistent with the theory of reasoned action, which holds that an individual’s intention influences whether they engage in a given behavior. It also depends on their attitude and the influence of their social environment, which can either positively or negatively affect an individual’s behavior [63,64]. Therefore, the women will have a positive attitude toward carrying out that behavior (ANC utilization) because they believe practicing BPCR will result in a positive outcome (i.e., ANC use). Our findings suggest that women’s attitudes toward BPCR practice have been predisposed to happen, amended, impacted, and changed due to participating in a six-month community-based HEI program [53] and ANC utilization. In addition, women who received HEI from intervention tend to have good knowledge of ANC, a favorable attitude, good health-seeking behavior, and information on the importance of ANC and, thus, may utilize ANC better.

Furthermore, several studies have demonstrated that women who have poor knowledge of ANC are often less prepared for delivery and complications and, consequently, often postpone seeking appropriate ANC services [65,66,67]. In addition, women more knowledgeable about ODSs communicate more effectively with health care providers (HCPs). Other researchers argued that women who are well-informed about ODSs have a greater probability of being prepared for childbirth and complications, which makes them more likely to utilize skilled ANC services [65,66,67]. Similar results were reported from the studies in Western Jamaica [67] and Sunyani Municipality, Ghana [66]. 

The HEI has increased the utilization of eight or more ANC visits between the two groups. The highest proportion of women who utilized at least one ANC visit might be due to the awareness created in mothers’ groups led by WDT leaders. However, the decreased number of eight or more ANC visits might be related to long distances from the nearest health facility, poor road conditions, and a lack of access to transportation [68]. According to another study conducted in Ethiopia, mothers are more likely to have more ANC follow-ups when there is adequate availability and access to ANC supplements, near distance from facilities, facilities readiness to offer skilled care, and availability of skilled HCPs [69,70]. However, women’s perceptions of ANC services as being ineffective or of poor quality may contribute to the low frequency of ANC visits. More efforts are needed in Ethiopia to achieve the WHO’s recently revised ANC standards on a positive pregnancy experience (WHO 2016) [46]. 

The finding that HEI significantly increases HFD utilization is similar to the findings of previous studies conducted in Alimosho Lagos [32] and Sokoto [29] states of Nigeria, western Kenya [71], and Nepal [72]. The reason might be that women who received HEI tend to possess good health-seeking behavior and are aware of the benefits of MHSs [73,74,75]. This study yielded results resembling real-world settings where flawless program attendance is rare. We found that HFD utilization improved significantly in the intervention group, confirming our hypothesis. Community health workers have appeared as a focal topic in worldwide discussions on upgrading primary healthcare systems over the last decade [76]. Evidence supports including these workers in delivering preventative maternal, newborn, and child health (MNCH) interventions like malaria prevention, breastfeeding promotion, basic infant care, health education, and psychosocial support [77]. Using a well-trained, community-based health worker corps to mobilize preventive health measures has shown promising results in decreasing maternal and neonatal mortality, especially in developing countries; yet, most of the existing literature emphasizes door-to-door rather than group-based HEI delivery models [71,78]. Group-based HE could be more effective than door-to-door HE in minimizing costs and time spent traveling from home to home to provide counseling on MHSs. In addition, small women’s groups build on this prevailing community and HF infrastructure to assist the most vulnerable mothers in remote rural poor communities. The small women’s groups significantly improve HFD utilization with increased assistance, supervision, and mentorship. 

However, a meta-analysis of seven cRCTs conducted in resource-limited settings (India, Malawi, Bangladesh, and Nepal) established a lack of intervention effects on HFD utilization [79]. Though data comparability is constrained by trial setting, design, and program structure variations, we found that the intervention arm had a significantly higher likelihood of HFD utilization. Similarly, our finding agrees with a previous Chamas study in rural western Kenya. These findings demonstrate the potential of our intervention to improve HFD utilization by integrating available infrastructure and community structures in contexts such as Ethiopia.

However, the HEI did not significantly improve PNC utilization. This finding agrees with studies performed in South Sudan [31] and Latin America [35]. Our qualitative study has identified several barriers to PNC use, such as home delivery, lack of awareness of PNC services and schedules, and sociocultural beliefs, and our intervention could not address socio-cultural barriers [68]. In this study area, the community members do not allow mothers in the postpartum period and newborns to leave the home due to sociocultural beliefs that the mother and newborn may be exposed to evil spirits. Due to these beliefs, they might not utilize PNC from HFs. Similar findings were documented in studies conducted in different settings [80,81]. Emerging evidence recommends that, besides identifying and overcoming financial barriers to MHS, initiatives to address sociocultural barriers may provide a compelling incentive for families to access competent care for delivery and early PNC at health facilities [82].

In the context of recently revised guidelines by the WHO on PNC for a positive postpartum period experience [83], more effort is required in Ethiopia to achieve the recommended number of PNC follow-ups. However, it is possible to guarantee increased coverage of PNC by providing SBA at a HF and adapting the community-based HEI by including resistance community members to promote PNC utilization in first-level facilities in low-resource contexts.

cRCTs are suitable when randomizing is not likely at the individual level or the intervention makes sense for a whole group or naturally occurring clusters [72,84]. When designing and analyzing public health research interventions, a cRCT is appropriate for assigning identifiable clusters or groups [85]. According to the CONSORT 2010 guidelines, a parallel cRCT is suitable where there is a possibility of accidental information contamination between two arms [86]. In our study, information cross-contamination can occur when mothers from one village interact with women from a different cluster. Various opportunities exist for social mixing or engagement in rural societies through travel or migration between comparator and treatment clusters; similarly, inhabitants of control clusters might be indirectly involved in intervention endeavors or, more likely, have informal conversations with intervention arm participants. As a result, residents of the comparator village may receive fundamental information about health messages supplied to the treatment villages. The most frequent challenge of information cross-contamination is the intervention effect’s dilution across two arms [72,84]. The following measures were considered to reduce information contamination between study groups: A buffer zone was constructed using four *kebeles* between the comparator and treatment clusters. This was accomplished by utilizing a map of all the districts. Before implementing the intervention, we allocated a midwife to address any difficulties or concerns about the HEI procedure from outside the research area. In addition, HEWs hired from a specific cluster made the cRCT possible and suitable to minimize such information contamination.

During the execution of the intervention components, we encountered some difficulties. Due to cultural taboos, women were unwilling to report their pregnancy status and underwent an HCG test during the early stages of pregnancy. However, this had no significant impact on our research findings. 

Randomization is believed to remove selection bias and produce similar results regarding unmeasurable and measurable confounders. However, this assumption might not hold in cRCTs, mainly if insufficient clusters are available. There is a considerable chance of a baseline covariate imbalance between the study groups when only a few clusters are available [41,42,43]. In this situation, it is often recommended to use multivariable analysis to account for the effect of baseline potential confounders or covariate imbalances and to evaluate the covariates for modification of the intervention effect [87,88]. In light of this, we have evaluated the effect modification and considered the measured sources of confounding in this analysis. The majority of covariates were similar in both study groups. Some of them, however, revealed a significant imbalance between the study groups, such as women’s occupation, use of mass media, husband’s occupation, wealth quintile, previous history of neonatal death, last pregnancy planned, facing health problems during the pregnancy, road access, receiving model family training, availability of transport at the individual level and place of residence, cluster-level mass media use, cluster-level distance to the nearest health facility, and cluster-level poverty at the community level. These imbalances were corrected using multilevel and multivariable analysis. We also assessed whether logical and plausible covariates influenced the intervention’s effectiveness. Since there was no statistically significant interaction term in the model, it proved that there was no statistically significant intervention effect modification by imbalanced confounders. Thus, we ruled out the possibility of effect modification; our findings were unaffected by covariate effect modification and were exclusively due to our intervention effect [87].

The ICC value indicated that belonging to *kebeles* accounted for 22.35% of the variability in ANC use, 21.88% in HFD use, and 10.76% in PNC use. The ICC value is more than 5% in all cases, which suggests that multilevel analysis is a method of choice [55,89]. The units of analysis in conventional ordinary regression methods are regarded as independent observations. The coefficient of regression standard errors may be overestimated if groupings are not considered, which could lead to an overestimation of statistical significance. Unable to account for the effect of clustering in the analysis stage, it will likely affect the standard errors or coefficients of higher-level determinants. The impacts of group-level variables are confused with the effects of group dummies in a fixed-effects ordinary model, making it impossible to distinguish between impacts arising from observed and unobserved group characteristics. However, in a multilevel random effects model analysis, the effects of both types of variables can be estimated successfully [55].

Two characteristics define randomized trials as the gold standard: randomization and double-blinding [88]. Because of the nature of the intervention, we could not mask (blind) the study participants or the research team; however, we masked data collectors. This would not, however, eliminate bias, which might result in an overestimation or underestimation of the intervention effect. Due to the open-label nature of the intervention and the use of the women’s self-reports for data collection, our findings may be impacted by information bias. Although difficult to measure, women’s awareness of their exposure status to intervention will likely influence their self-response answers to the knowledge and MHSU questions, resulting in information bias. There is a chance that personally related variables like ANC, HFD, and PNC utilization will be purposefully over-reported or underreported (social desirability bias). As a result, the magnitude of the HEI effect may have been overestimated.

Another drawback was that we only had one intervention follow-up period of six months, so we could not determine whether ANC and HFD utilization were sustained over more prolonged times, particularly in the treatment group. Though our qualitative study identified several barriers to MHSU, our intervention cannot address distance from health facilities, costs associated with MHSU, waiting time to obtain MHSs, road accessibility, the transportation arrangements during unpredictable labor, the needs of poor mothers, sociocultural barriers, or supply-side barriers [68]. Further, to guarantee that the intervention has long-lasting effects in the research setting, the remaining effects are also not assessed through an after-project study conducted a few periods after the project is finished. 

In this study, we have adjusted for numerous potential confounders using a multivariable modified Poisson regression model. However, confounding from unmeasured sources or residual confounding like lack of information about the level of husband involvement in MHSs, HCP skills, health facility service trust by women, women’s knowledge of MHSs, level of service availability, and accessibility between two arms, HCPs-to-population ratio, and compassionate, caring, and respectful maternal health care implementation status were not measured during data collection. Thus, these variables’ residual or unmeasured confounding influences cannot be excluded.

Furthermore, we were unable to evaluate the events of every enrolled woman during the time we collected the data due to several factors, such as 24 mothers moving, 13 stillbirths, 17 abortions, and two maternal deaths. This caused missing outcome data, which violates the principle of randomization. In principle, randomization ensures that the two arms are comparable or balanced for known and unknown confounders only when women are initially randomized. When a portion of one or both groups’ membership is gone, the two groups can no longer be considered balanced. This bias may cause the intervention effect to be underestimated or overestimated.

Furthermore, such attrition reduces the sample size and threatens this study’s statistical power, making it incapable of detecting the actual effect of the HEI or more prone to type II error [88]. In both groups, the percentage of mothers lost to follow-up was similar (4.98% in the treatment group compared to 5.87% in the comparator group), which is inconsequential in our case. In addition, the fact that we lost merely 4.8% of the sampled women is consistent with the norm of a smaller than five percent loss to follow-up, which is thought to represent a low risk of bias in cRCTs and has no discernible impact on ITTA results [88]. In addition, we performed a post-hoc assessment of power and found that for both ANC and HFD, the statistical power was 100%, sufficient to identify the effects of the intervention. Thus, despite the abovementioned limitations, the trial’s findings are adequate to develop effective MHSU strategies, programs, or policies.

This study has various strengths. To minimize duplication, we recorded the research protocol on ClinicalTrials.gov with the reference number NCT05865873 after getting ethical permission. To determine the temporal relationship, we utilized a cRCT study design with comparator and treatment groups, a vital epidemiological design for establishing causality between exposure and outcome. Because the sample size of this study was large, we could identify the HEI’s impacts on outcomes. As a result, the findings apply to all women in similar study settings, and they are critical in formulating suitable policy measures for an efficient and successful promotion of ANC and HFD utilization. Research from Nigeria [29,30,33] and Ghana [34] also reported consistent findings, indicating that this conclusion might also hold for developing nations at comparable levels of socioeconomic development, cultural context, and access to healthcare services. For the scalability and sustainability of the intervention, we strengthened the existing community structure of WDTs rather than establishing additional structures. 

## 5. Conclusions

Our six-month community-based HEI significantly increased the utilization of skilled ANC and HFD while not improving the use of PNC. Thus, expanding the HEI with certain modifications, such as mobilizing more stable and active community members, addressing demand and supply-side concerns related to distance from health facilities, costs associated with MHSU, waiting time to obtain MHSs, road accessibility, transportation arrangements during unpredictable labor, the needs of poor mothers, sociocultural barriers, quality of services, and skilled HCPs, as well as repeated or longer HEI. Furthermore, PNC utilization is very low during the early postnatal period; adaptation of HEI must be prioritized, and attention should be given to the inclusion of husbands, more socioculturally adherent community groups about postpartum taboos of hiding delivered mothers, and home-based visits to increase PNC utilization.

## Figures and Tables

**Table 1:** Effect of HEI on ANC utilization in the northern zone of Sidama regional state, Ethiopia, 2023 (N = 1070).

Variables	Antenatal Care		CRR (99% CI)	ARR (99% CI)
	Utilized	Not Utilized		
	N (%)	N (%)		
Individual level determinants				
Study group				
Comparator	355 (67.0)	175 (33.0)	Ref	Ref
Treatment	489 (90.6)	51 (9.4)	1.35 (1.19, 1.54)	1.32 (1.12, 1.56) *
Women’s occupation				
Housewife	599 (75.3)	196 (24.7)	Ref	Ref
Farmer	39 (79.6)	10 (20.4)	1.04 (0.88, 1.21)	1.06 (0.87, 1.28)
Government employee	105 (93.8)	7 (6.3)	1.24 (1.11, 1.37)	1.05 (0.95, 1.17)
Merchant	101 (88.6)	13 (11.4)	1.17 (1.03, 1.34)	1.04 (0.94, 1.16)
Husband occupation				
Government employee	109 (93.2)	8 (6.8)	Ref	Ref
Merchant	435 (79.7)	111 (20.3)	0.87 (0.79, 0.94)	1.02 (0.91, 1.13)
Farmer	300 (73.7)	107 (26.3)	0.79 (0.70, 0.88)	1.01 (0.89, 1.13)
Use of mass media				
No	375 (70.4)	158 (29.6)	Ref	Ref
Yes	469 (87.3)	68 (12.7)	1.04 (1.01, 1.06)	1.10 (0.98, 1.24)
Wealth quintile				
Lowest	187 (87.8)	26 (12.2)	Ref	Ref
Second	161 (74.9)	54 (25.1)	0.86 (0.74, 1.01)	0.95 (0.84, 1.06)
Middle	146 (68.2)	68 (31.8)	0.78 (0.64, 0.95)	0.86 (0.74, 0.99) *
Fourth	154 (72.0)	60 (28.0)	0.82 (0.69, 0.96)	0.85 (0.75, 0.97) *
Highest	196 (91.6)	18 (8.4)	1.02 (0.93, 1.15)	0.96 (0.86, 1.08)
Previous history of neonatal death				
No	817 (79.2)	215 (20.8)	Ref	
Yes	27 (71.1)	11 (28.9)	0.90 (0.65, 1.25)	1.04 (0.82, 1.32)
Last pregnancy planned				
No	175 (61.4)	110 (38.6)	Ref	Ref
Yes	669 (85.2)	116 (14.8)	1.38 (1.25, 1.52)	1.32 (1.18, 1.49) *
Faced health problems during the pregnancy				
No	749 (77.3)	220 (22.7)	Ref	Ref
Yes	95 (94.1)	6 (5.9)	1.21 (1.12, 1.31)	1.24 (1.14, 1.34) *
Road access				
Inaccessible	582 (76.8)	176 (23.2)	Ref	Ref
Accessible	262 (84.0)	50 (16.0)	1.09 (0.98, 1.21)	0.98 (0.86, 1.13)
Received model family training				
No	503 (75.1)	167 (24.9)	Ref	Ref
Yes	341 (85.3)	59 (14.8)	1.13 (1.04, 1.23)	1.07 (0.98, 1.16)
Accessibility of transport				
No	405 (74.4)	139 (25.6)	Ref	Ref
Yes	439 (83.5)	87 (16.5)	1.12 (1.03, 1.20)	1.03 (0.95, 1.12)
Community-level determinants				
Place of residence				
Rural	386 (48.3)	414 (51.7)	Ref	Ref
Urban	154 (57.0)	116 (43.0)	0.91 (0.72, 1.14)	0.85 (0.69, 1.04)
Cluster-level mass media use				
Low	521 (78.6)	142 (21.4)	Ref	Ref
High	323 (79.4)	84 (20.6)	1.01 (0.84, 1.20)	0.95 (0.82, 1.11)
Cluster-level distance to reach the nearby health facility				
Big problem	239 (81.6)	54 (18.4)	Ref	Ref
Not big problem	605 (77.9)	172 (22.1)	0.96 (0.77, 1.18)	1.09 (0.94, 1.28)
Cluster-level poverty				
Low	667 (80.6)	161 (19.4)	Ref	Ref
High	177 (73.1)	65 (26.9)	0.90 (0.73, 1.11)	0.99 (0.82, 1.20)

*: significant association (*p* < 0.01); Ref: reference group; CI: confidence interval; ARR: adjusted risk ratio; CRR: crude risk ratio.

**Table 2:** Effect of HEI on eight-or-more ANC utilization in the northern zone of Sidama regional state, Ethiopia, 2023 (N = 1070).

Variables	Eight or More Antenatal Care		CRR (99% CI)	ARR (99% CI)
	Utilized	Not Utilized		
	N (%)	N (%)		
Study group				
Control	116 (21.9)	414 (78.1)	Ref	Ref
Intervention	204 (37.8)	336 (62.2)	1.81 (1.03, 3.17)	1.51 (1.03, 2.22)

Note: Variables adjusted in the models were women’s occupation, mass media, husband’s occupation, use of wealth quintile, previous history of neonatal death, last pregnancy planned, faced health problems during the pregnancy, road access, received model family training, availability of transport, place of residence, cluster-level mass media use, place of residence, and cluster-level poverty; Ref: reference group; CI: confidence interval; ARR: adjusted risk ratio; CRR: crude risk ratio.

**Table 3:** Effect of HEI on HFD utilization among women of reproductive age in the Northern zone of Sidama region, Ethiopia, 2023 (N = 1070).

Variables	Health Facility Delivery		CRR (95% CI)	ARR (99% CI)
	Utilized	Not Utilized		
	N (%)	N (%)		
Individual level determinants				
Study group				
Comparator	327 (61.7)	203 (38.3)	Ref	Ref
Treatment	456 (84.4)	84 (15.6)	1.37 (1.21, 1.55)	1.24 (1.06, 1.46) *
Women’s occupation				
Housewife	554 (69.7)	241 (30.3)	Ref	Ref
Farmer	30 (61.2)	19 (38.8)	0.88 (0.73, 1.07)	0.89 (0.70, 1.13)
Government employee	104 (92.9)	8 (7.1)	1.33 (1.19, 1.48)	1.08 (0.93, 1.27)
Merchant	95 (83.3)	19 (16.7)	1.21 (1.06, 1.37)	1.11 (0.97, 1.28)
Husband occupation				
Government employee	108 (92.3)	9 (7.7)	Ref	Ref
Merchant	395 (72.3)	151 (27.7)	0.79 (0.72, 0.87)	0.97 (0.89, 1.06)
Farmer	280 (68.8)	127 (31.2)	0.75 (0.65, 0.85)	1.01 (0.88, 1.16)
Use of mass media				
No	340 (63.8)	193 (36.2)	Ref	Ref
Yes	443 (82.5)	94 (17.5)	1.29 (1.12, 1.49)	1.17 (0.99, 1.38)
Wealth quintile				
Lowest	166 (77.9)	47 (22.1)	Ref	Ref
Second	149 (69.3)	66 (30.7)	0.89 (0.75, 1.07)	0.99 (0.81, 1.23)
Middle	131 (61.2)	83 (38.8)	0.79 (0.64, 0.96)	0.90 (0.73, 1.11)
Fourth	147 (68.7)	67 (31.3)	0.87 (0.75, 1.02)	0.95 (0.77, 1.17)
Highest	190 (88.8)	24 (11.2)	1.12 (0.99, 1.27)	1.02 (0.85, 1.22)
Previous history of neonatal death				
No	760 (73.6)	272 (26.4)	Ref	Ref
Yes	23 (60.5)	15 (39.5)	0.83 (0.64, 1.07)	0.95 (0.70, 1.28)
Last pregnancy planned				
No	163 (57.2)	122 (42.8)	Ref	Ref
Yes	620 (79.0)	165 (21.0)	1.06 (1.03, 1.08)	1.29 (1.14, 1.46) *
Faced health problems during the pregnancy				
No	696 (71.8)	273 (28.2)	Ref	Ref
Yes	87 (86.1)	14 (13.9)	1.37 (1.22, 1.53)	1.22 (1.08, 1.37) *
Road access				
Inaccessible	536 (70.7)	222 (29.3)	Ref	Ref
Accessible	247 (79.2)	65 (20.8)	1.13 (0.99, 1.29)	1.03 (0.89, 1.18)
Received model family training				
No	462 (69.0)	208 (31.0)	Ref	Ref
Yes	321 (80.2)	79 (19.8)	1.16 (1.04, 1.28)	1.06 (0.95, 1.18)
Availability of transport				
No	369 (67.8)	175 (32.2)	Ref	Ref
Yes	414 (78.7)	112 (21.3)	1.17 (1.06, 1.29)	1.09 (0.97, 1.20)
Community-level determinants				
Place of residence				
Rural	600 (75.0)	200 (25.0)	Ref	
Urban	183 (67.8)	87 (32.2)	0.89 (0.77, 1.05)	0.90 (0.78, 1.01)
Cluster-level mass media use				
Low	491 (74.1)	172 (25.9)	Ref	Ref
High	292 (71.7)	115 (28.3)	0.97 (0.81, 1.14)	0.93 (0.81, 1.07)
Cluster-level distance to nearest health facility				
Big problem	244 (83.3)	49 (16.7)	Ref	Ref
Not big problem	539 (69.4)	238 (30.6)	0.83 (0.69, 1.01)	0.92 (0.79, 1.08)
Cluster-level poverty				
Low	624 (75.4)	204 (24.6)	Ref	Ref
High	159 (65.7)	83 (34.3)	0.87 (0.75, 1.01)	0.95 (0.82, 1.08)

*: significant association (*p* < 0.01); Ref: reference group; CI: confidence interval; ARR: adjusted risk ratio; CRR: crude risk ratio.

**Table 4:** Effect of HEI on PNC utilization in the northern zone of Sidama regional state, Ethiopia, 2023 (N = 1070).

Variables	Postnatal Care		CRR (99% CI)	ARR (99% CI)
	Utilized	Not Utilized		
Study Group				
	N (%)	N (%)		
Comparator	276 (52.1)	254 (47.9)	Ref	Ref
Treatment	353 (65.4)	187 (34.6)	1.26 (1.04, 1.54)	1.15 (0.89, 1.48)

Note: Variables adjusted in the models were women’s occupation, mass media, husband’s occupation, use of wealth quintile, previous history of neonatal death, last pregnancy planned, faced health problems during the pregnancy, road access, received model family training, availability of transport, cluster-level mass media use, place of residence, cluster-level distance to nearest health facility, and cluster-level poverty; Ref: reference group; CI: confidence interval; ARR: adjusted risk ratio; CRR: crude risk ratio.

## Data Availability

This published article and its Supplementary Information files include all data generated or analyzed during this study.

## Supplementary Materials

The following supporting information can be downloaded at: https://www.mdpi.com/article/10.3390/healthcare12101045/s1, File S1: CONSORT 2010 checklist (DOC); File S2: Study questionnaire (DOC); File S3: Some important information in the methods and result section (DOC); File S4: Stata data set (dta). References [90,91,92,93,94,95,96,97] are cited in the supplementary materials.

## Abbreviations

ANC: Antenatal Care; AIC: Akaike’s Information Criterion; ARRs: Adjusted Risk Ratios; BPCR: Birth Preparedness and Complication Readiness; BIC: Bayesian Information Criterion; CI: Confidence Interval; EDHS: Ethiopian Demographic and Health Survey; HCP: Health Care Provider; HCG: Human Chorionic Gonadotropin; HEI: Health Education Intervention; HF: Health Facility; HFD: Health facility Delivery; HEW: Health Extension Worker; ICC: Intra-Cluster Correlation Coefficient; ITTA: Intention to Treat Analysis; IRB: Institutional Review Board; LBs: Live Births; MHS: Maternal Health Service; MHSU: Maternal Health Service Utilization; MMR: Maternal Mortality Rate; NRR: Non-response Rate; ODS: Obstetric Danger Sign; ODK: Open Data Kit; PNC: Postnatal Care; cRCT: Cluster Randomized Controlled Trial; SBA: Skilled Birth Attendant; SCT:Social Cognitive Theory; SD: Standard Deviation; SSA: Sub-Saharan Africa; WDA: Women Development Army; WDT: Women Development Team; WHO: World Health Organization; VIF: Variance Inflation Factor.
