# Supplementary material for: Community-Based Health Education Led by Women’s Groups Significantly Improved Maternal Health Service Utilization in Southern Ethiopia: A Cluster Randomized Controlled Trial
Source: Healthcare (Basel). 2024 May 18;12(10):1045. doi: 10.3390/healthcare12101045 (PMC11121210; doi:10.3390/healthcare12101045)
Supplement: Supplementary file 1 [file healthcare-12-01045-s001.zip › File S1.pdf]

CONSORT 2010 checklist of information to include when reporting a randomised trial*

Section/Topic	Item No	Checklist item	Reported on page No
**Title and abstract**			
	1a	Identification as a randomised trial in the title	
	1b	Structured summary of trial design, methods, results, and conclusions (for specific guidance see CONSORT for abstracts [1, 2])	2
**Introduction**			
Background and objectives	2a	Scientific background and explanation of rationale	
	2b	Specific objectives or hypotheses	4-6
**Methods**			
Trial design	3a	Description of trial design (such as parallel, factorial) including allocation ratio	
	3b	Important changes to methods after trial commencement (such as eligibility criteria), with reasons	7
Participants	4a	Eligibility criteria for participants	
	4b	Settings and locations where the data were collected	
Interventions	5	The interventions for each group with sufficient details to allow replication, including how and when they were actually administered	7
Outcomes	6a	Completely defined pre-specified primary and secondary outcome measures, including how and when they were assessed	12 and 13
	6b	Any changes to trial outcomes after the trial commenced, with reasons	N/A
Sample size	7a	How sample size was determined	8
	7b	When applicable, explanation of any interim analyses and stopping guidelines	N/A
Randomisation:			
Sequence generation	8a	Method used to generate the random allocation sequence	9
	8b	Type of randomisation; details of any restriction (such as blocking and block size)	
Allocation	9	Mechanism used to implement the random allocation sequence (such as sequentially numbered	

concealment mechanism		containers), describing any steps taken to conceal the sequence until interventions were assigned	
Implementation	10	Who generated the random allocation sequence, who enrolled participants, and who assigned participants to interventions	9
Blinding	11a	If done, who was blinded after assignment to interventions (for example, participants, care providers, those assessing outcomes) and how	9
	11b	If relevant, description of the similarity of interventions	12
Statistical methods	12a	Statistical methods used to compare groups for primary and secondary outcomes	
	12b	Methods for additional analyses, such as subgroup analyses and adjusted analyses	
**Results**			
Participant flow (a diagram is strongly recommended)	13a	For each group, the numbers of participants who were randomly assigned, received intended treatment, and were analysed for the primary outcome	10 and 18
	13b	For each group, losses and exclusions after randomisation, together with reasons	18
Recruitment	14a	Dates defining the periods of recruitment and follow-up	
	14b	Why the trial ended or was stopped	18
Baseline data	15	A table showing baseline demographic and clinical characteristics for each group	20-24
Numbers analysed	16	For each group, number of participants (denominator) included in each analysis and whether the analysis was by original assigned groups	
Outcomes and estimation	17a	For each primary and secondary outcome, results for each group, and the estimated effect size and its precision (such as 95% confidence interval)	24-29
	17b	For binary outcomes, presentation of both absolute and relative effect sizes is recommended	
Ancillary analyses	18	Results of any other analyses performed, including subgroup analyses and adjusted analyses, distinguishing pre-specified from exploratory	24-29
Harms	19	All important harms or unintended effects in each group (for specific guidance see CONSORT for harms[28])	17
**Discussion**			
Limitations	20	Trial limitations, addressing sources of potential bias, imprecision, and, if relevant, multiplicity of analyses	34
Generalisability	21	Generalisability (external validity, applicability) of the trial findings	34-35

Interpretation	22	Interpretation consistent with results, balancing benefits and harms, and considering other relevant evidence	30-33
**Other information**			
Registration	23	Registration number and name of trial registry	ClinicalTrials.gov with registration number NCT05865873
Protocol	24	Where the full trial protocol can be accessed, if available	
Funding	25	Sources of funding and other support (such as supply of drugs), role of funders	Hawassa University and Sidama region president office

*We strongly recommend reading this statement in conjunction with the CONSORT 2010 Explanation and Elaboration [3] for important clarifications on all the items. If relevant, we also recommend reading CONSORT extensions for cluster randomised trials, [4] non-inferiority and equivalence trials [5], non-pharmacological treatments [6], herbal interventions [7], and pragmatic trials [8]. Additional extensions are forthcoming: for those and for up to date references relevant to this checklist, see www.consort-statement.org.

References

1. Hopewell S, Clarke M, Moher D, Wager E, Middleton P, Altman DG, et al. CONSORT for reporting randomised trials in journal and conference abstracts. *Lancet* 2008;371:281-3.
2. Hopewell S, Clarke M, Moher D, Wager E, Middleton P, Altman DG, et al. CONSORT for reporting randomized controlled trials in journal and conference abstracts: explanation and elaboration. *PLoS Med* 2008;5:e20.

3. Moher D, Hopewell S, Schulz KF, Montori V, Gøtzsche PC, Devereaux PJ, et al. CONSORT 2010 Explanation and Elaboration: updated guidelines for reporting parallel group randomised trials. *BMJ* 2010;340:c869.
4. Campbell MK, Elbourne DR, Altman DG. CONSORT statement: extension to cluster randomised trials. *BMJ* 2004;328:702-8.
5. Piaggio G, Elbourne DR, Altman DG, Pocock SJ, Evans SJ. Reporting of noninferiority and equivalence randomized trials: an extension of the CONSORT statement. *JAMA* 2006;295:1152-60.
6. Boutron I, Moher D, Altman DG, Schulz KF, Ravaud P. Extending the CONSORT statement to randomized trials of nonpharmacologic treatment: explanation and elaboration. *Ann Intern Med* 2008;148:295-309.
7. Gagnier JJ, Boon H, Rochon P, Moher D, Barnes J, Bombardier C. Reporting randomized, controlled trials of herbal interventions: an elaborated CONSORT statement. *Ann Intern Med* 2006;144:364-7.
8. Zwarenstein M, Treweek S, Gagnier JJ, Altman DG, Tunis S, Haynes B, et al. Improving the reporting of pragmatic trials: an extension of the CONSORT statement. *BMJ* 2008;337:a2390
