# Supplementary material for: Community-Based Health Education Led by Women’s Groups Significantly Improved Maternal Health Service Utilization in Southern Ethiopia: A Cluster Randomized Controlled Trial
Source: Healthcare (Basel). 2024 May 18;12(10):1045. doi: 10.3390/healthcare12101045 (PMC11121210; doi:10.3390/healthcare12101045)
Supplement: Supplementary file 1 [file healthcare-12-01045-s001.zip › File S2.pdf]

1. ANNEXES

Annex I: Information sheet

Hawassa University

College of Medicine and Health Sciences

School of Public Health

Project title: Improving maternal health service utilization among women of reproductive age in North Zone of Sidama National Regional State, Ethiopia

Hello! My name is ---. I am a data collector in a study being conducted about maternal health service utilization under the College of Medicine and Health Sciences of Hawassa University. We are conducting a study to improve maternal health service utilization in your community.

Goal of the study

The objective of this study is to improve maternal health service utilization. The study will be conducted for six months. After this baseline survey, selected mothers in some villages will be provided health education regarding maternal health topics.

At the end of intervention, we will test or check how well the education has assisted to improve MHSU in your villages by comparing to other villages that did not obtain education. The data obtained in this study will be valuable to prepare the education regarding maternal health topics.

Significance of the study and method of participant selection

The aim of this study is to generate information about the maternal health service utilization among women of reproductive age in North Zone of Sidama region. The study may help public health planners, stakeholders, policy makers, responsible body, and others to take actions based on the finding. The study comprises various intimate and private questions. We randomly selected you to help us by participating in this study as the data you offer us will helpful in averting maternal and child death and illness. Interview will take not more than 40 minutes.

Risks and discomfort

We assure you that there is no risk or harm in participation of this study. The study comprises various friendly and private questions. However, you may refuse to respond any of the queries if you feel uncomfortable.

Benefits

Your involvement will assist us to find out more regarding maternal health issue in the villages and to provide education to women in your communities. If this study revealed that the education is useful in increasing maternal health by improving proper use of MHS, the government and other stakeholders will be able to improve maternal health in other communities in this setting and other part of the country.

Incentives

There is no incentive or payment for participating in this research. Nonetheless, we will thank you very much for your involvement.

Confidentiality

All information will be kept confidentially. Name of a participant will not be written or specified. Your privacy will also be protected and no one shall know your response.

Right to withdraw or refuse

You have full right whether or not to participate in this study. You may respond to all questions or you may not answer to the questions you don't want to or you may quit your participation totally at any time you want. You can ask any questions which is not clear for you. Moreover, we assure you that refusing to participate will not affect your future treatment at the health institution or elsewhere in any way.

Who to contact

You can ask any questions which are not clear for you now or later. If you wish to ask queries later, you may contact: Amanuel Yoseph, Hawassa University, Telephone: 0915683578.

Annex II: Informed Consent

I have been requested to take part in the research and read the foregoing information, or it has been read to me in the language I comprehend and understand all condition stated above. I have had the opportunity to ask questions about it and any questions I have been asked have been answered to my satisfaction. I consent voluntarily to be a participant in this study and understand that I have the right to withdraw from the interview at any time without in anyway affecting my right.

Are you willing to participate in this study?

Yes ☐ No ☐

If "Yes", study participant's ID……………………Signature…………….Date…………..

And proceed with the interview.

If "No" ....thank you and end.

Name of the principal investigator: Amanuel Yoseph

Cell phone Number - +251-915683578 E mail: amanuelyoseph45@gmail.com

Name of interviewer____________________ Signature__________

Data of interviewer (Ethiopia calendar) _____/_____/_____

Result of interview: 1. Complete 2. Refused 3. Partially complete 4. Respondent not available

Cheeked by supervisor: Name______________ Signature_________ Data___/_____/____

Annex III: English version questionnaire

Hawassa University

College of Medicine and Health Sciences

School of Public Health

Questionnaire designed to study the maternal health service utilization among women of reproductive age in rural North Zone of Sidama National Regional State, Ethiopia.

PART I: General Information

01. Name of Woreda: ____________

02. Name of Kebele: ________________

03. Name of Gote __________________

04. House number ____________________

05. Individual Id __________________

06. Date of interview: ____________time interview started __________ ended at ____________

07. Respondent available on:

1. 1st visit.
2. 2nd visit.
3. 3rd visit.

08: Data collection phase 1. Baseline (before intervention) 2. Endline (post-intervention)

09: Post-intervention data collection 1. Study participants from intervention group
2. Study participants from control group

Module I: Socio-demographic characteristics of study participants

Instruction: First, tell interviewee that you are going to ask her questions about herself. Second, properly explain to the study participant each question to elicit genuine response from the respondents. Then, request her to correctly respond to the questions. Please write the responses based on the respondents genuine responses (in field to be filled by data collectors).

S.no	Questions	Response categories	Skip to	Remark
101	What is your age (in completed years)?	__________ years		
102	To which ethnic group do you belong?	1. Sidama<br>2. Amhara<br>3. Oromo<br>4. Gurage<br>5. Wolayita<br>88.Other,specify_____		
103	What is your religious affiliation?<br>(encircle the response)	1. Protestant Christian<br>2. Orthodox Christian<br>3. Catholic<br>4. Muslim<br>5.Other (specify) __________		
104	What is your highest educational level you have completed?	1. Cannot read and write<br>2. Can read and write only<br>3. Primary education(1-8)<br>4. Secondary education(9-12)<br>5. College diploma<br>6. College/university degree and above		
105	What is your occupation?	1. Housewife<br>2. Farmer<br>3. Governmental employee<br>4. Merchant		

		5. Housemaid<br>6. Other (specify)………………		
106	What is your marital status?	1. Not ever married……………<br>2. Married<br>3. Divorced<br>4. Separated<br>5. Widowed<br>6. Cohabiting	Q110	
107	What is your husband‘s age in complete years?	________________		
108	What is your husband‘s occupation?	1. Governmental employee<br>2. Merchant<br>3. Farmer<br>4. Daily labourer<br>5. NGO employee<br>6. Private organization employee<br>7. Other (specify)………………		
109	What is your husband‘s educational status?	1. Cannot read and write<br>2. Can read and write only<br>3. Primary education(1-8)<br>4. Secondary education(9-12)<br>5. College diploma<br>6. College/university degree and above		
110	What is your family size? (In number)	_________		
111	Which social media do you use?	1. Listens the radio<br>2. Watches the television<br>3. Reads the newspaper<br>4. All<br>5. Others		

Module II: Household wealth index of study participants

S.no	Questions	Response categories	Skip to	Remark
201	What is the main source of drinking water for members of your household?	1. Piped water<br>2. Tube well or Borehole<br>3. Protected well<br>4. Unprotected well<br>5. Protected spring<br>6. Unprotected spring<br>7. Rain water<br>8. Surface water (River/dam)<br>9. Lake/pond/stream/canal<br>10. Bottled water		
202	What is the main source of water used by your household for other purposes such as cooking and handwashing?	1. Piped water<br>2. Tube well or Borehole<br>3. Protected well<br>4. Unprotected well<br>5. Protected spring<br>6. Unprotected spring<br>7. Rain water<br>8. Surface water (River/dam)<br>9. Lake/pond/stream/canal<br>10. Bottled water		
203	Where is that water source located?	1. In own dwelling<br>2. In own yard/plot<br>3. Elsewhere		
204	How long does it take to go there, get water, and come back?	Minutes . . . .<br>998. Don’t know		
205	In the past two weeks, was the water from this source not available for at least one full day?	1. Yes<br>0. No<br>998. Don’t know		

206	Do you do anything to the water to make it safer to drink?	1. Yes<br>0. No<br>998. Don't know		
207	What kind of toilet facility do members of your household usually use?<br><br>IF NOT POSSIBLE TO DETERMINE, ASK PERMISSION TO OBSERVE THE FACILITY.	1. Flush or pour flush toilet<br>2. Ventileted improved pit latrine<br>3. Pit latrine with slab<br>4. Pit latrine without slab/open pit<br>5. Compositing toilet<br>6. Bucket toilet<br>7. Hanging toilet<br>8. No facility/bush/field		
208	Do you share this toilet facility with other households?	1. Yes<br>0. No		
209	Including your own household, how many households use this toilet facility?	______________________<br>998. Don't know		
210	Where is this toilet facility located?	1. In own dwelling<br>2. In own yard/plot<br>3. Elsewhere		
211	What type of fuel does your household mainly use for cooking?	1.Electricity<br>2. Liquefied petroleum gas<br>3. Natural gas<br>4.Biogas<br>5.Keresone<br>6. Charcoal<br>7. Wood<br>8. Straw/shrubs/grass<br>9. Agricultural crop<br>10. Animal dung<br>11.No food cooked in household		
212	Is the cooking usually done in the house, in a separate building, or outdoors?	1. In the house<br>2. In a separate building		

		3. Outdors<br>4. Others (specify)………………		
213	Do you have a separate room which is used as a kitchen?	1. Yes<br>0. No		
214	Who is the owner of the house?	1. Me<br>2. Rental<br>3. Family<br>4. Relative<br>5. Others (specify)…………..		
215	How many rooms in this household are used for sleeping?	-------------------------		
216	Main material of the roof of the house?	1. Natural roofing (no roof, mud, and sod)<br>2. Rudimentary roofing (rustic mat/plastic shee, reed/bamboo, wood planks,and cardboard)<br>3. Finished roofing (metal/corrugated iron, wood, calamine/cement, ceramic tiles, roofing shingles)		
217	Main material of the floor of the house?	1. Natural floor (Earth/sand, dung)<br>2. Rudimentary floor (wood planks,and palm/bamboo)<br>3. Finished floor (parquet or polished wood, vinyl or asphalt strips/ plastic tiles, cement, ceramic tiles, carpet)		
218	Main material of the wall of the house?	1. Natural walls (no walls, cane/palm/trunks/bamboo/ree, dirt)<br>2. Rudimentary walls (bamboo		

		with mud, stone with mud, uncovered adobe, plywood, cardboard, and reused wood)<br>3. Finished floor (cement, stone with lime/cement, bricks, cement blocks, covered adobe, wood planks/shingles)		
219	Does this household own any livestock, herds, other farm animals, or poultry?	1.Yes<br>0.No	 222	
220	How many of the following animals does this household own?<br>IF NONE, RECORD '00'.<br>IF 95 OR MORE, RECORD '95'.<br>IF UNKNOWN, RECORD '98'.<br>a) Milk cows, oxen or bulls?<br>b) Other cattle?<br>c) Horses/donkeys/mules?<br>d) Camels?<br>e) Goats?<br>f) Sheep?<br>g) Chickens/poultry?<br>h) Beehives?	 a) Milk cows, oxen or bulls………<br>b) Other cattle………………….<br>c) Horses/donkeys/mules……….<br>d) Camels……………………..<br>e) Goats………………………<br>f) Sheep………………………….<br>g) Chickens/poultry……………..<br>h) Beehives…………………….		
221	Do you have separate rooms for cattle?	1.Yes<br>0.No		
222	Does any member of this household own any agricultural land?	1.Yes<br>0. No	 224	
223	How many hectares of agricultural land do members of this household own?	 --------------------------hectares		
224	Does your household have:<br>a) Electricity?<br>b) A radio?	Yes No<br>a) Electricity……..1 0<br>b) Radio…………..1 0		

	c) A television?<br>d) A non-mobile phone?<br>e) A computer?<br>f) A refrigerator?<br>g) A table?<br>h) A chair?<br>i) A bed with cotton/sponge/spring mattress?<br>j) An electric mitad?<br>k) A kereson lamp/pressure lamp?	c) Television………1 0<br>d) Non-mobile phone.1 0<br>e) Computer…………1 0<br>f) Refrigerator………1 0<br>g) Table………………1 0<br>h) Chair………………1 0<br>i) Bed with cotton/sponge/spring mattress…………..1 0<br>j) Electric mitad…….1 0<br>k) Kereson lamp/pressure lamp……………….1 0		
225	Does any member of this household own:<br>a) A watch?<br>b) A mobile phone?<br>c) A bicycle?<br>d) A motocycles/scooter?<br>e) An animal drawn cart?<br>f) A car/truck?<br>g) A boat with motor?<br>h) A bajaja?	Yes No<br>a) Watch . . . . 1 0<br>b) Mobile phone . . . . . 1 0<br>c) Bicycle. . . . . 1 0<br>d) Motocycles/scooter. . 1 0<br>e) Animal drawn cart . . .1 0<br>f) Car/truck . . . . .1 0<br>g) Boat with motor . . . . . 1 0<br>h) Bajaja . . . . ……..1 0		
226	Does any member of this household have a bank account?	1. Yes<br>0. No		
227	Does any member of this household have a microfinanceaccount?	1. Yes<br>0. No		

Module III: Reproductive history of study participants

S.no	Questions	Response categories	Skip to	Remark
301	What was your age when you were first married?	____________________<br>998. I don't know		
302	What was your age at your first pregnancy?	____________________<br>998. I don't know		
303	Have you ever become a pregnant?	0. No<br>1. Yes	3	
304	If yes for question 303, how many times?	---------------------------		
305	Have you ever faced abortion?	0. No<br>1. Yes	307	
306	If yes for question 305, how many times?	----------------------------		
307	Have you ever delivered a child?	0. No<br>1. Yes	311	
308	If yes for question 306, how many times?	-----------------------------		
309	How many of them were live births?	------------------------------		
310	How many of them were delivered at home?	---------------------------		
311	Infection during the current pregnancy (e.g., UTI, periodontal disease)	0. No<br>1. Yes		
312	Do you have any family history of hypertension?	0. No<br>1. Yes	3	
313	If Yes to Q312, who?	1. Father<br>2. Mother<br>3. Sister<br>4. Grand mother		

		5. Other (specify)………………..		
314	Have you ever experienced death of fetus or stillbirth (fetal death at or after 28 weeks of pregnancy)?	0. Yes<br>1. No<br>998. I don‘t remember		
315	Have you ever experienced death of neonate (age less than seven days)?	0. Yes<br>1. No<br>998. I don‘t remember	end	
316	If yes to Q 314, how many times?	______________		
317	If yes to Q 314, where was the neonate born?	1. Home<br>2. Health institution<br>3. Other (specify) _________		

Module IV: Maternal health service utilization

S.no	Questions	Response categories	Skip to	Remark
401	Was your last pregnancy planned?	1. Yes<br>0. No	405	
402	If ‘No’ to Q401, what was reason?	1. Lack of modern contraceptives<br>2. Failure of contraceptive method<br>3. Others (specify)…………………		
403	If ‘No’ to Q401, don’t want at all the last pregnancy?	1. Yes<br>0. No		
404	If ‘No’ to Q401, do you want after months of delivery?	1. Yes<br>0. No		
405	Did you face any health problem during your the last pregnancy?	1. Yes<br>0. No	407	
406	If ‘Yes’ to Q 405, what were they?<br>(Do not read the choices)	1. Excessive vaginal bleeding<br>2. A high grade fever<br>3. Bad smelling vaginal discharge<br>4. Convulsions<br>5. Severe headache<br>6. Face/ hand swelling<br>7. Persistent vomiting<br>8. High blood pressure<br>9. Other (specify)_________		
407	Did you visit a health facility during your last pregnancy?	1. Yes<br>0. No	409	
408	If yes to Q 407, reason for visit?	1. Pregnancy related health problem<br>2. Health problems not related to pregnancy<br>3. For antenatal care		

		4. Other (specify) ------------------		
409	Did you obtain ANC visit during your last pregnancy?	1. Yes<br>0. No<br>998. Don't remember	42<br>8<br>42<br>8	
410	If yes to Q409, how many times did you obtain ANC?	______________________________<br>998.Don't know/remember	41<br>2	
411	Mention chronologically in how many months did you obtain ANC visits?	1st ANC ……………….months<br>2nd ANC ………………months<br>3rd ANC………………..months<br>4th ANC……………….months<br>999. Don't know/remember		
412	From where did you receive ANC service?	1. Government hospital<br>2. Private hospital<br>3. Faith based charity 16ospital<br>4. Health centre<br>5. Private clinic<br>6. Faith based charity clinic<br>7. Health post<br>8. Other (specify) _________________		
413	Why did you prefer the above stated health facility (Q412)?	1. Close to my house<br>2. Competent health worker<br>3. Other (specify) _________		
414	Who provided you ANC in the last pregnancy?	1. Doctors<br>2. Nurse<br>3. Midwifes<br>4. Health officers<br>5. Health extension workers		

		6. Traditional birth attendant<br>7. Other (specify)____________		
415	Did you receive any tetanus toxoid (TT) injection during your last ANC visits?	1. Yes<br>0. No<br>998. Don't remember		
416	If 'Yes' to Q415, how many times?	____________________		
417	Did you receive any laboratory test during your last ANC visits?	1. Yes<br>0. No<br>998. Don't remember		
418	If 'Yes' to Q417, what tests?	1= Blood (Blood groups)<br>2= Blood (Syphilis test)<br>3= Stool<br>4= Urine<br>5= Other (Specify)………………………		
419	Did you receive any health message during your last ANC visits?	1. Yes<br>0. No<br>998. Don't remember	42<br>8<br>42<br>8	
420	If 'Yes' to Q419, what messages?	1= Breastfeeding<br>2= Danger Signs Maternal<br>3= Birth Planning<br>4= Facility Delivery<br>5= Danger Signs New-born<br>6= Basic Care New Born<br>8= Other (Specify)………………………	42<br>1	
421	Which danger signs of were you informed about?<br>(Don't read the choices)	l= Severe headache<br>2= Blurry Vision<br>3= Reduced or absent foetal movement<br>4= High blood pressure		

		5= Oedema of the face<br>6= Oedema of the hands & feet<br>7= Convulsions<br>8= Vaginal bleeding<br>9= Lower abdominal pain<br>l0=None of above		
422	Were you informed where to go during danger signs problems stated above (Q421)?	1. Yes<br>0. No<br>998. Do not remember		
423	Where you informed about where to deliver during your last baby?	1. Yes<br>0. No<br>998. Do not remember	42<br>5<br>42<br>5	
424	If yes to Q 423, where were you recommended to deliver?	1. Home<br>2. Health post<br>3. Health centre<br>4. Private clinic<br>5. Faith based charity clinic<br>6. Government hospital<br>7. Private hospital<br>8. Faith based charity hospital<br>9. Others specify………………		
425	Were you informed about who should attend you during delivery?	1. Yes<br>0. No<br>998. Do not remember	42<br>7<br>42<br>7	
426	If ‘Yes’ to Q 425, who was recommended to attend your delivery?	1. Doctors<br>2. Nurse/Midwifes/health officers<br>3. HEWs		

		4. Traditional birth attendant<br>5. Other (specify)__________		
427	Were you informed to prepare the following items before your delivery date?	1. Transport<br>2. Save money<br>3. Identify blood donor<br>4. Nothing		
428	If you didn't ANC visits, can you tell me the reasons?<br>(Multiple answer is possible)<br>(Do not read the choice)	1.No or little Knowledge about ANC<br>2. No health problem encountered<br>3. Health institution is too far from my home<br>4. Long waiting time<br>5. Poor handling by health care providers<br>6. Lack of transportation<br>7. Lack of time to go to health institution<br>8. Other (specify)---------------------		
429	Did you plan to deliver in health facility?	1. Yes<br>0. No<br>998. Don't remember	43<br>1	
430	If no to Q429, can you tell me the reasons?<br>(Multiple answer is possible)<br>(Do not read the choice)	1.No or little Knowledge about institutional delivery<br>2. No health problem encountered<br>3. Health institution is too far from my home<br>4. Poor handling by health care providers<br>5. Lack of transportation<br>6. Other (specify)---------------------		
431	Before your date of delivery for your last pregnancy did you or your family prepared the following?	1. Transport<br>2. Save money<br>3. Identify appropriate health facility<br>4. Identify blood donor<br>5. Identify skilled attendant<br>6. Nothing		
432	Where did you give birth to your last child?	1. Home<br>2. Health post	44<br>2	

		3. Health centre<br>4. Private clinic<br>5. Faith based charity clinic<br>6. Government hospital<br>7. Private hospital<br>8. Faith based charity hospital<br>9. Others specify………………		
433	If your last delivery is in health institution, when did you go to the health institution?	1. At the beginning of labour<br>2. 6-12 hours after the beginning of labour<br>3. 13-18 hours after the beginning of labour<br>4. 19-24 hours after the beginning of labour<br>5. Other specify __________		
434	If your last delivery is in health institution, what was the mode of delivery?	1. Spontaneous vaginal delivery<br>2. Instrumental delivery<br>3. Caesarean section<br>4. Other (specify) __________		
435	Who conducted or assisted you in the last delivery?	1. Doctors<br>2. Nurse<br>3. Midwife<br>4. Health officer<br>5. Health extension worker<br>6. Traditional birth attendant (TBA)<br>7. Other (specify)___________		
436	Did anyone other than health care providers assist you with the delivery of last baby?	1. Yes<br>0. No		
437	If 'Yes' to Q436, who assisted you with the delivery?<br><br>Anything else?	1. Mother<br>2. Mother-in law<br>3. Sister<br>4. Sister-in law<br>5. Other member of in-laws home		

	RECORD ALL RESPONSES	6. Other member of fathers<br>7. Other relatives<br>8. Neighbour<br>9. Other specify……………………		
438	Were any of the following procedures performed at the time of delivery?	1. Blood transfusion<br>2. Fluid<br>3. Injection<br>4. Episiotomy<br>5. Received tablet Misoprostol		
439	If injection provided. What was the injection for?<br>(Do not read the choice)<br><br>RECORD ALL RESPONSES	1= Speed up labour<br>2 = Reduce bleeding<br>3= Stop convulsions<br>4=Other (Specify)<br>999=Don't Know		
440	For how long were you in labour during the last delivery?	1. Less than 12 hours<br>2. 12- 24 hours<br>3. 25- 36 hours<br>4. 37 – 48 hours<br>5. More than 48 hours		
441	What was the condition of the baby at birth in the last delivery?	1. Born alive<br>2. Still birth (born died)<br>3. Born alive but died immediately		
442	If responded at home to Q432, why did you prefer to deliver at home?<br>(Don't read the choice)	1. The labour was short<br>2. No nearby health facility<br>3. The service is not available in the nearby health facility<br>4. Lack of money for transport<br>5. Poor handling by health professionals<br>6. Prefer to deliver in the presence of relatives<br>7. Fear of manipulation (like episiotomy)<br>8. Lack of privacy in the health institutions		

		9. I didn‘t know the importance of health facility delivery<br>10.Opinions of (husband, neighbours, other community members)<br>11. Other (specify) ____________		
443	Have you encountered any health problems during labour in the last delivery?	1. Yes<br>0. No<br>999. I don‘t remember	45<br>6	
444	If yes to Q 443, what were they?<br>(Do not read the choices)	1 = Excessive Vaginal bleeding<br>2 = Foul smelling discharge<br>3 = High grade fever<br>4 = Baby’s hand or feet come first<br>5= Baby bad position/ mal presentation<br>6= Prolong labour (> l2 Hours)<br>7= Retained placenta/placental expelled late<br>8 = Torn uterus<br>9= Prolapsed cord<br>10 = Cord around the neck<br>11= Convulsions<br>l2 = Perineal tear<br>l3= None of above	45<br>6	
445	Did you seek any care or treatment for any of these/this complication(s)?<br>Probe: Any care/treatment receive at home	1. Yes<br>0. No	45<br>6	
446	If ‘Yes’ to Q445, where did you seek care/treatment for these/this complication(s)?<br>RECORD ONLY ONE	1=Home<br>2=Health post<br>3=Health centre<br>4= Private clinic		

	ANSWER- WHERE WOMAN OR FAMILY FIRST SOUGIIT CARE/TREATMENT	5=Faith based charity clinic<br>6=Government hospital<br>7=Private hospital<br>8=Faith based charity hospital<br>9=Others specify………………		
447	If ‘Yes’ to Q445, who provided this care/treatment?<br>RECORD ONLY ONE ANSWER	1=Doctors<br>2=Nurse<br>3=Midwife<br>4=Health offer<br>5=Health extension worker<br>6=Traditional birth attendant (TBA)<br>7=Family<br>8=Relative<br>9=Neighbour<br>10=Other (specify)___________		
448	Were you referred to any other place for care/treatment of complication(s)?<br>PROBE. CARE INSIDE/OUTSIDE THE HOME?	1. Yes<br>0. No	 45<br>1	
449	If ‘Yes’ to Q448, where were you referred?	1. Health post<br>2. Health centre<br>3. Hospital<br>4. Prayer<br>5. Other specify……………………..		
450	Did they tell you the reasons for referral?	1. Yes<br>0. No	 45<br>5	
451	If ‘Yes’ to Q449, why were you told to seek treatment at this	1 = No surgery equipment<br>2 = High blood pressure		

	than other place?<br>RECORD ALL RESPONSES	3 = For better treatment<br>4 = Doctor unavailable<br>5 = No arrangement for blood transfusion<br>6 = Baby's upward position<br>7 = Some part of baby came out<br>8 =Baby Stool in womb<br>9 = cervix did not open<br>10 = Other specify<br>999 = Don't know		
452	Did you go to this place?	1. Yes<br>0. No<br>999. Don't remember	45<br>5	
453	If 'Yes' to Q452, how long did you wait to seek care for this complication?	________________		
454	If 'Yes' to Q449, did anybody call you after you want to the referred place, to see how you were doing?	1. Yes<br>0. No<br>999. Don't remember		
455	Why did you not go to the referred place?	1 = Woman didn't think necessary<br>2 = Husband /family didn't think necessary<br>3 = Facility too far<br>4 = No transport<br>5 = No child care<br>6 = too expensive<br>7 = Services are poor quality<br>8 = Didn't know where to go<br>9 = No time to go<br>10 = Money not easily available<br>I l = Other (Specify)<br>99 = Don't know		

456	Did you use the transport you identified during pregnancy?	1. Yes<br>0. No		
457	Did you use the money that you saved during pregnancy?	1. Yes<br>0. No		
458	Have you encountered any health problems during the first 6 weeks of post-partum?	1. Yes<br>0. No<br>999. I don‘t remember	46<br>0	
459	If ‘Yes’ to Q 455, what were they?<br>(Do not read the choices)	1 = Excessive vaginal bleeding<br>2 = Foul smelling discharge<br>3 = High fever<br>4 = Inverted nipples<br>5 = Tetanus<br>6 = Retained placenta<br>7 = Severe abdominal pain<br>8 = Convulsions<br>9 = Engorged breasts<br>10 = None of above		
460	Did you get medical check-up or PNC after your last delivery within 42 days?	1. Yes<br>0. No<br>999. I don‘t remember		
461	If ‘Yes’ to Q461, when did you get it for the first time after delivery?	1. After ______ hours<br>2. After ______ days<br>3. After ______ weeks		
462	If ‘Yes’ to Q461, how many times did you obtain PNC within 42 days?	______________________<br>998.Don’t know/remember		
463	Where did you get the check-up for the first time?	1= Home<br>2=Health post<br>3=Health centre<br>4=Hospital<br>5=Others specify………………		

464	What additional services did you get?	1. Child vaccination<br>2. Family planning methods<br>3. Counselling about breast feeding<br>4. Others specify _____________		
465	From where did you receive PNC service?	1. Government hospital<br>2. Private hospital<br>3. Faith based charity 26ospital<br>4. Health centre<br>5. Private clinic<br>6. Faith based charity clinic<br>7. Health post<br>8. Other (specify) _________________		
466	Who provided you PNC in the last childbirth?	1=Doctors<br>2=Nurse<br>3=Midwifes<br>4=Health officers<br>5=Health extension workers<br>6=Traditional birth attendant<br>7=Other (specify)____________		
467	If you didn't PNC visits, can you tell me the reasons? (Multiple answer is possible) (Do not read the choice)	1.No or little Knowledge about ANC<br>2. No health problem encountered<br>3. Health institution is too far from my home<br>4. Long waiting time<br>5. Poor handling by health care providers<br>6. Lack of transportation<br>7. Lack of time to go to health institution<br>8. Other (specify)---------------------		
468	What was your husband's attitude towards MHSU?	1. Positive<br>2. Negative<br>3. Don't know		
469	Who is the decision maker in	1. Both myself and my husband		

	your HH to seek care from modern health institution?	2. My husband<br>3. My self<br>4. Other (specify) ______________		
470	Is road accessible to near health facility?	1. Yes<br>0. No		
471	Have you taken model household training?	1. Yes<br>0. No		

Module V: Knowledge and attitudes of study participants towards MHSU

1. Knowledge of study participants towards MHSU

S.no	Questions	Response categories	Skip to	Remark
	**Knowledge questions for ANC**			
501	Do you know about the ANC service?	1. Yes	503	
502	If yes to Q 501, is ANC important for mothers and fetus positive health outcome?	0. No<br>1. Yes		
503	When does the first ANC visit is recommended?	0. Greater than 16 weeks<br>1. Less than 16 weeks		
504	Do you know total number of ANC visits recommended for the pregrant women throughout her preganancy period?	0. Less than 4<br>1. Four and more		
505	Where is the appropriate place for the first ANC visit?	0. Health post<br>1. Helth center or hospital		
506	Does a pregnant woman need to undergo the laboratory test during her ANC visit?	0. No<br>1. Yes		
507	Does a pregnant woman need to be provided the essential drugs during her ANC visit?	0. No<br>1. Yes		

508	Does a pregnant woman need to be provided the counseling during her ANC visit?	0. No<br>1. Yes		
	**Knowledge questions for institutional delivery**			
509	Do you know about the institutional delivery service?	0. No<br>1. Yes		
510	If yes to Q 501, is institutional delivery care important for mothers and newborn positive outcome?	0. No<br>1. Yes		
511	Which woman is recommended to give birth at HFs?	0. High risk<br>1. All		
512	Which place is safe for child delivery?	0. Home<br>1. HFs		
513	Which provider is skilled for child delivery?	0. TBA, relative, friend<br>1. Health professional		
514	Does a woman die due to the complications happen during child birth?	0. No<br>1. Yes		
515	Does a SBA at HFs can prevent maternal deaths that happen during the child birth?	0. No<br>1. Yes		
	**Knowledge questions for PNC**			
516	Do you know about the PNC service?	0. No<br>1. Yes		
517	If yes to Q 501, is PNC important for mothers and neonetus positive health outcome?	0. No<br>1. Yes		
518	Do you know total number of PNC visits recommended for the women throughout her pospartum period?	0. Less than 3<br>1. Three and more		
519	Where is the appropriate place for the first PNC visit?	0. Health post<br>1. Helth center or hospital		

520	Does a pregnant woman need to be provided the counseling during her PNC visit?	0. No<br>1. Yes		
521	Does a woman die due to the complications happen during postpartum period?	0. No<br>1. Yes		
522	Does a PNC at HFs can prevent maternal deaths that happen during the postpartum period?	0. No<br>1. Yes		

2. **Attitude of study participants towards MHSU**

<u>Tool to assess client attitude towards MHSU</u>

1-Strongly disagree; 2- Disagree; 3- Neutral; 4- Agree; 5- Strongly agree

S.no	Questions	1	2	3	4	5
	**Attitude questions for ANC**					
523	Your attitude about the ANC is important for mothers' positive health outcomes?					
524	Your attitude about the first ANC visit should be obtained in less than 16 weeks?					
525	Your attitude about the four ANC visits is adequate for uncomplicated pregnancy?					
526	Your attitude about the health post is an inappropriate place for the first ANC visit?					
527	Your attitude about the pregnant woman needs to use the laboratory test during her ANC visit?					
528	Your attitude about the pregnant woman needs to be providing the essential drugs during her ANC visits?					
529	Your attitude about the pregnant woman needs to be					

	providing counsel during her ANC visits?					
530	Your attitude about the women's reasons not to go to a health facility for ANC is mainly because of high opportunity cost?					
531	Your attitude about the women's reasons not to go to a health facility for ANC because HCPs don't treat them respectfully?					
	**Attitude questions for institutional delivery**					
532	Your attitude about institutional delivery care is important for mothers' positive health outcomes?					
533	Your attitude about all women is recommended to give birth at HFs?					
534	Your attitude about the HFs is safe for child delivery?					
535	Your attitude about the HCPs being skilled professionals for child delivery?					
536	Your attitude about the woman dying due to the complications happens during childbirth?					
537	Your attitude about the SBA at HFs can prevent maternal deaths that happen during childbirth?					
538	Your attitude about the importance of having a plan on possible pregnancy complications?					
539	Your attitude about the delivery being attended by male HCPs isn't shameful?					
540	Your attitude about the delivery on the delivery bed isn't shameful in the labour ward?					
541	Your attitude about the women doesn't go to a health facility for delivery is due to high opportunity cost?					
542	Your attitude about the women doesn't go to a health facility for delivery due to HCPs doesn't treat them respectfully?					
	**Attitude questions for PNC**					

543	Your attitude about the PNC is important for mothers' positive health outcomes?					
544	Your attitude about the 3 PNC visits is adequate for the women with uncomplicated delivery?					
545	Your attitude about the health post is an inappropriate place for the first PNC?					
546	Your attitude about the delivered woman needs to be provided the counsel during her PNC visit?					
547	Your attitude about the woman dying due to the complications happens during the postpartum period?					
548	Your attitude about the PNC visits in HFs can prevent maternal deaths that happen during the postpartum period?					
549	Your attitude about the women doesn't go to a health facility for PNC is due to high opportunity cost?					
550	Your attitude about the women doesn't go to a health facility for PNC is due to HCPs don't treat them respectfully?					

Module VI: Socio-cultural predictors

S.no	Questions	Response categories	Skip to	Remark
601	Can you tell me any socio-cultural beliefs that affect MHSU in your community?<br><br>(Do not read the choices)<br><br>RECORD ALL RESPONSES	1. Availability and influence of traditional healers<br>2. Availability and influence of spiritual healers<br>3. Availability and influence of traditional birth attendants<br>4. Holy water<br>5. Fear of using MHS<br>6. Peer influence<br>7. Knowledge of community towards MHSU<br>8. Husband influence<br>9. Father and mother in law influence<br>10. Other (specify)………….		

Model VII: Perceived quality of MHSU

Tool to assess client perceived quality of antenatal care

1-Very poor; 2- Poor; 3- Neutral; 4- Good; 5- Very good

	ITEMS	1	2	3	4	5
1	Your perception about the friendliness shown towards you by the HCPs in the ANC					
2	Your perception about the patience shown towards you when you did not cooperate with HCPs					
3	Your perception about the promptness of the attention given by the HCPs when you needed it					
4	Your perception about the enough time with HCPs during ANC visit					
5	Your perception about the way your privacy was maintained by the HCPs in the ANC room					
6	Your perception about the willingness of the HCPs to discuss about your concerns					
7	Your perception about the your involvement in decision making					
8	Your perception about the fairness of waiting time in the ANC visit					
9	Your perception about the overall cleanliness of the HF					
10	Your perception about the help you received from the health care workers to take care of fetus and yourself (ex: maintaining your cleanliness)					
11	Your perception about the adequacy of information given to you on ANC benefits					
12	Your perception about the adequacy of information given on expected date of delivery and gestational age					
13	Your perception about the adequacy of information given to you to identify danger signals					

14	Your perception about the skills of the HCPs to identify and manage health issues					
15	Your perception about adequacy of information received to clarify any issues you had					
16	Your perception about the Cleanliness of the ANC room					
17	Your perception about adequacy of information received on the nutrition					
18	Your perception about the information on Tetanus toixed vacination					
19	Your perception about the information on blood test					
20	Your perception about the information on urine test					
21	Your perception about the information on weight, height measurement					
22	Your perception about the information and blood pressure measurement					
23	Your perception about an appointment clarity					

Tool to assess mother perceived quality of institutional delivery care

1-Strongly disagree; 2- Disagree; 3- Neutral; 4- Agree; 5- Strongly agree

S.no	Questions	1	2	3	4	5
1	I think there is good waiting area in the nearby health facility					
2	I think there is good maternity ward's toilets in the nearby health facility					
3	Hand washing and shower is available in the nearby health facility					
4	Infrastructures like electricity, and water is available in the nearby health facility					
5	It is easy to get maternity ward starting from the gate					
6	The health care providers in the nearby clinic are good at labor and delivery care					
7	The health care providers in the nearby clinic are good at newborn care					
8	The health care providers in the nearby clinic are good at pain management					
9	How long does it take to reach to the nearby health facility on foot?	________hrs				
10	Prompt transport service is available from home to the nearby health facility	1. Yes<br>0. No				
11	Delivery service charge in the nearby health facility	1. High<br>2. Moderate<br>3. Low				

Tool to assess client perceived quality of institutional postnatal care

1-Very poor; 2- Poor; 3- Neutral; 4- Good; 5- Very good

	ITEMS	1	2	3	4	5
1	Your perception about the friendliness shown towards you by the HCPs in the postnatal ward					
2	Your perception about the patience shown towards you when you did not cooperate with HCPs					
3	Your perception about the promptness of the attention given by the HCPs when you needed it					
4	Your perception about the availability of pain relief during the postpartum period					
5	Your perception about the way your privacy was respected by the HCPs in the postnatal ward					
6	Your perception about the willingness of the HCPs to discuss about your concerns					
7	Your perception about the way HCPs treated your family members					
8	Your perception about the help given for the initiation of breast feeding in the labour room					
9	Your perception about the help you received from the HCPs to take care of your baby					
10	Your perception about the help you received from the health care workers to take care of yourself (ex: maintaining your cleanliness)					
11	Your perception about the adequacy of information given to you on taking care of the baby					
12	Your perception about the adequacy of information given on proper method of breast feeding					
13	Your perception about the adequacy of information to identify					

	danger signals following delivery, for the mother & the baby					
14	Your perception about the skills of the HCPs to identify and manage health issues of your baby					
15	Your perception about the skills of the HCPs to identify and manage health issues in relation to you					
16	Your perception about adequacy of information received to clarify any issues you had					
17	Your perception about the Cleanliness of the ward					
18	Your perception about the Cleanliness of the toilets & washrooms					
19	Your perception about adequacy of space in the postnatal ward					
20	Your perception about the availability of adequate facilities in the ward in relation to the number of patients					
21	Your perception about adequacy of delivery beds in the labour room					
22	Your perception about the availability of adequate numbers of HCPs to assist you					
23	Your perception about the ability to get some rest in the postnatal ward (without the interferences such as light, noise, ward activities)					

Module IIX: Knowledge of ODS during pregnancy, childbirth and postpartum periods

S.no	Questions	Response categories	Skip to	Remark
801	Please tell me what complications may occur during pregnancy that needs rnedical care?<br><br>(Do not read the choices)<br><br>Anything else?<br><br>RECORD ALL RESPONSES	l= Severe headache<br>2= Blurry vision<br>3= Reduced or absent foetal movement<br>4= High blood pressure<br>5= Oedema of the face<br>6= Oedema of the hands & feet<br>7= Convulsions<br>8= Vaginal bleeding<br>9= Lower abdominal pain<br>l0=None of above		
802	Now, please tell me what are the complications in women during child birth that need medical treatment?<br><br>(Do not read the choices)<br><br>Anything else?<br><br>RECORD ALL RESPONSES	1 = Excessive vaginal bleeding<br>2 = Foul smelling discharge<br>3 = High grade fever<br>4 = Baby's hand or feet come first<br>5= Baby bad position/ mal presentation<br>6= Prolong labour (> l2 Hours)<br>7= Retained placenta<br>8 =Torn uterus<br>9= Prolapsed cord<br>10 = Cord around the neck<br>11= Convulsions<br>l2 = Perineal Tear<br>l3= None of above		
803	Now, please tell me what are the complications in women immediately child birth that need medical treatment?<br><br>(Do not read the choices)<br>Anything else?	1 = Excessive vaginal bleeding<br>2 = Foul smelling discharge<br>3 = High fever<br>4 = Inverted nipples<br>5 = Tetanus<br>6 = Retained placenta		

	RECORD ALL RESPONSES	7 = Severe abdominal pain<br>8 = Convulsions<br>9 = Engorged breasts<br>10 = None of above		

Module IX: Practices of BPCR

S.no	Questions	Response categories	Skip to	Remark
901	During pregnancy and before the delivery, did you or your family make any plans for the birth?	l= Yes<br>0= No<br>999= Don't Remember	906	
902	If yes to Q901, which plans did you or your family makes for the birth of (NAME)?<br>(Do not read the choices)<br>Anything else?<br>RECORD ALL RESPONSES	l= Identify proper closer HFs for childbirth<br>2= Identify SBA for deliver<br>3= Save money and material resources<br>4= Identify proper transport for delivery<br>5= Identify and fixing the compatible blood group givers		
903	If identified facility for delivery. Where did you plan to give birth to (NAME)?<br>(Do not read the choices)	1. Home<br>2. Health post<br>3. Health centre<br>4. Hospital<br>5. Others specify………………		
904	If identified provider for delivery. Who did you select as the birth attendant?<br>(Do not read the choices)	1. Doctors<br>2. Nurse/Midwifes<br>3. Health extension workers<br>4. Traditional birth attendant<br>5. Other (specify)___________		
905	If identified transport. Did you use the transport you identified during pregnancy?	1. Yes<br>0. No		
906	If saved money. Did you use the money that you saved during pregnancy?	1. Yes<br>0. No		

Thank you for your participation!!!

2. QA’MISO

Qa’miso I: MASHALAQQETE QOOLA

Hawaasi Yuniversite

HIKKIMINNUNNA FAYYIMMATE SAYINSE KOLLEEJE

Dagoomu Fayyimmate Rosi Mine

Xiinxallote umo: DIRINSA ILATE DEERRIRA NOO AMUWI FAYYIMMARA UYINANNI OWAANTE WOYYEESSA Sidaamu Dagoomu Qoqqowu Mootimma, Itophiya

AYIDDE KEEREHO!! Su’ma’ya __________________ yinannie, ani xiinxallote taje gamba aseemoho.

Fayyimmate rosi horo amuwu fayyimmate owaante horora meentu fayyimmate qodhaano horonsi’ratenni afi'rino rosi marichi horo abbinoronna amuwu fayyimma aana luphi yiino qeechi maati amuwaho fayyimmate owaante horo aana dagoomu giddo yaanno qaru koorinniiti.

XIINXALLOTE GOOFIMARCHU MIXONKE

Amuwu reyo dagankera ikko kalqete qarraati.Qajeelino amuwi fayyimma owaataasine shiimu baatooshshinni calla ikkado lifinxenni amuwu xissonna reyo ajishanno qarra tirate hayyooti. Xiinxallote akeeki qaru guulchu hedo amuwaho rosichonsa luphi assatenna fayyimansa agarate. Xiinxaallo xiinxaallinannihu 6 aganaati.Tenne kaimu taje gamba assinihu gedensaanni, dooramino amuwira ikkado fayyimmate rosicho amuwu fayyimma daafira uyinanni. Ikkado rosicho uynihu gedensaanni, awuwu rosicho afi'rihu gedensaanni, fayyimmansara marichi egennonna rosicho afi'ri yaatenna ikkado fayyimmate rosicho afi'rinokki amuwira may badooshshi no yaateetinna heewisiisateeti rosicho afi'rinohanna afi'rinokki amuwa.

Taje tini horontanni amuwu fayyimma daafira callaati.

XIINXAALLOTE BEEQQORA DOORROONNI HAYYO

Xiinxaallote beeqqora doorroonni hayyo sassaete. Konni daafira ati beeqqaancho ikkootta.Borri-xa'mote aattanke dawaro amatenna daaimu xissoranna reyo ittisate aatenke taje luphiima

kaa’litanonke.Sumuu yaattae ikkiro, borri-xa’mo nabbawa hanafeemmo.Taje aattata mittu mannino fojo horontanni dikullanni.Sumuu yaa giwittaro borri-xa’mo agure sa’’a dandaatta.Xiinxallo mereeho agurate qoossokki agarantinote.Wolu xiinxallaanchi nafa fojokki afa dindadaanno.

XIINXALLOTE BEEQQOOTTA DAAFIRA MITTE HULULO QARRI DINO

Xiinxallote beeqqootta daafira mite mexamme woy huluullo heedhoonkehe!

Huluullamittaro dee’ni yaa dandaatta.

.

Xiinxallote Horo

Xiinxallo amuwaho uytanno horo umikkihunni Amuwu fayyimmate horo maatiro afate, layinkihunni Amuwaho fayyimmate owaantera marichi badooshshi nooro afatenna awuwu godowara albaanni may qarri xaadannonsaro balaxe huwanyo heedhaansa gede assatenna rosichonsa buuxate.Qoleno amuwu fayyimmate owaantera may badooshshi nooronna awuwu fayyimmate owaantera hiittene amaati owaante afi’ra hasiissannoti yee badateeti. Anfe ka’niro taaltino amaale aatenna wole fayyimmate uurrinshsha tareessateeti.Ate beeqqo xiinxaallote uytanno kaa’lo ate qachira no awuwira fayyimmansa afaateeti.Ledotenno ikkado fayyimmate rosicho qachikkira uyineemo gede kaa’lannonke. Ikkado fayyimmate rosicho dagankenna gobbanke.

Xiinxallote beeqqo lainohunni baatooshshu dino.

Xiinxallote beeqqora baatooshshu dino ikkinohura, beeqqo assootta daafira lowo galati noe..

FOJJO LAINOHUNNI.

Wo’manti taje kulattaeti, lowo geeshsha fojo wolu mannira dikuleemmo .Qole ate su’ma horontanni diborreeseemmo.Horontanni dawarootta dawaro ayeno diafanno.

XIINXALLOTE BEEQQO ASSATTOHU SUMUU YIITTARONNA MEREEROHO XA’MO XA’ME GUDUMMOKKINO AGURATE QOOSSOKKI AGARANTINOTE!

Xiinxallote beeqqo assa hoogakki mittoreno amuwu afi’ranno owaante hooltannohetano di"ikitino/mereeroho dawarte gudittakkinni agurte ha'ra dandaatta.

Xa’mo heedhuhero woy xawinnokkiri nooro, Amunuel Yooseefiha Hawaasi yuniversitera loosaasincho xa tenne yannara xiinxallote aana xa’ma dandiitinanni

Bilibilu kiirosi: 0915683578.

Qa'miso II: MASHALAQQE AATE FAJJO

SUMUU YAATTAE? DANCHA FAJJOOTEKKI IKKIRO: EE ☐ Dee'ni ☐

Tumi-qolokki ‘’ Ee’’ ikkiro ... Xa’mote Sai.

Xa’mamaanchu malaate________________________________

‘’Dee’ni’’ ikkiro Uurrissi!!

Xiinxallaanchu su’ma: Amanuel Yoseph

Bilbilu kiiro - +251-915683578 E-maile: amanuelyoseph45@gmail.com

Xa’manchu su’ma___________________ malaate__________

Xa’minoonni barra Tophiyu kiiro garinni borreessi. Xa’maanchu Qaali-xamo _____/_____/_____

Xa’mote dawaro: 1. Wo’manka dawaroonni. 2. Sumuu diyitino 3. Gama maahoyye yite gama gibbinno. 4. Xa’mamaancho hooggino mine.

Illaallisaanchu su’ma:______________ malaate_________ barra__/_____/____

Qa’miso III: MASHALAQQETE QOOLA

Hawaasi Yuniversite

HIKKIMINNUNNA FAYYIMMATE SAYINSE KOLLEEJE

Dagoomu Fayyimmate Rosi Mine

Tini Borri-Xa’mo Qixxaabbinohu Itophiyu GiddoSidaamu Dagoomu Qoqqowu MootimmaAmuwu Ilate Dirinsa Keeraanchimma Buuxate Uynanninsa Owaante Aanaati

GAAMO I: Xaphoomu Taje

01. Woradu su’ma: ____________

02. Olluu su’ma: ________________

03. Qachumu su’ma __________________

04. Minu kiiro_____________________

05. Ayimmate Kaarde kiiro __________________

06. Xa’minoonni barra ____________Xa’mo hananfoonni yanna __________ Xa’mo gooffinno yanna.____________

07. Xa’mamaanchu towaanyo. Mashalaqqisaanchu qooxeessaho no:

1. Umikki towaanyo
2. Layinki towaanyo
3. Sayikki towaanyo.

08: Taje gamba assate gafa 1. Ikkito ikkate albaanni (before intervention) 2. Ikkito ikkite sa’eenna (post-intervention)

09: Ikkito ikkite sa’eenna gamba assinanni taje 1. Xiinxallote taji-mashalaqqisaano beeqqaano gaamo

2. Xiinxallote taji-mashalaqqisaano beeqqaanchiweelo gaamo

Mojule I: Mashalaqqisaanonniha Dagi-mannoomittete Heeshsho Gade

Biddissa: Umihunni, qaali-xamishsha assakkira balaxxe dawootto/a haja odeessi. Layinkimeeshsho, mashalaqqisaanokki halaalaancho xawishsha uytahe geeshsha tittirte dawoottore xawisi.Jeefoteno, mashalaqqisaanokki uytuhe taje garunni borreessite amadi ballo (Xiinxallaanchu taje gamba assanno ba're).

A.K	Xa'mo	Mashalaqqisaanote Tumo	Sai/ kubbi	Buuxo
101	Dirikki me''eho (xaphooma)?	__________ diro		
102	Hiikko ayiddeeti? / Hiikko mineeti?	1. Sidaama<br>2. Amaara<br>3. Oromo<br>4. Guraage<br>5. Wolayitta<br>6. woleno, xawisi____		
103	Ammanokki hiitteeti?<br>(dawarokki qoqqowi)	1. Pirotestaante /Kiristaana<br>2. Ortodokise / Kiristaana<br>3. Kaatoolike<br>4. Musiliime<br>5.Wolete? (xawisi) __________		
104	Jawiidihu rosikki deerri hiikkonneeti?	1.Nabbawanna borreessa didandeemmo/a<br>2. Nabbawanna borreessa calla dandeemmo/a<br>3. Umi dirimi rosaanchooti (1-8)<br>4. 2ki dirimi rosaanchooti (9-12)<br>5. Dippoloomu rosaanchooti<br>6. Digiretenna hakkuy aleeti		
105	Loosu Qeechi maati?	1. Minaama (mini ama)<br>2. Baatto loossi're galinoha<br>3. Mootimmate Loosaasincho		

		4. Daddalaancho<br>5. Gobbate giddoydi<br>6.Wolereno (Xawisi)		
106	Adhhammete Gara?	1. Leexa …………….<br>2. Adhaminoha<br>3. Adhe/ ite Tirroha<br>4. Baxxinoha<br>5. Shiidhinoha<br>6. Woleno (Xawisi) _______	X110	
107	Halaalaancho galtekki diro	_______________		
108	Galtekki loosi qeechi maati?	1. mootimmate loosaasinchooti<br>2. daddalaanchoho<br>3. baatto loosi're, saada ce'e galinoho<br>4. barru looso loose hee'rannoho<br>5. mootimma ikkitinokki (NGO) loosaasinchooti<br>6. hallanyu uurrinsha giddo loosanno<br>7. woleno, xawisi -------------------		
109	Galtekki rosi deerri mageeshshiho?	1.Nabbawanna borreessa dandaannokkiho<br>2. Nabbawanna borreessa calla dandaannoho<br>3. Umi dirimi rosaanchooti (1-8)<br>4. Layinki dirimi rosaanchooti (9-12)<br>5. Dippolooma ronsoho<br>6. Digirenna hakkuy ale		
110	Maatekki batinyi mageeshshiho? (kiirotenni)	________		

111	Hiikkonne dagoomi-tuqi-xaado horoonsi’ratta?	6. Macciishshinanni raadoone<br>7. La’nanni televishiine<br>8. Nabbambanni gaazeexa<br>9. Baalanka<br>10. woleno, xawisi		

Mojule II: Maatete Miccinna Miinji Mayimma Mashalaqqe

A.K	Xa’mubba	Response categories	Sai/kub bi	Buuxo
201	Maatekki aggannoha co’icha waa afidhannohu mamiinniiti?	1. Baambu waa<br>2. Tuubbote waa<br>3. Huxxaminoha balete waa<br>4. Huxxaminokkiha balete waa<br>5. Huxxinoonniha buete waa<br>6. Huxxaminokkiha burqanno waa<br>7. Xeenu waa<br>8. Daadanno waa (lagga/xashshuwa)<br>9. Garba /kooficho/ga’rinowaa<br>10. Hayilaandete waa		
202	Maatekki sagale qishi’ratenna anga hayishshi’rate horoonsidhanno waa hiikkiinni afidhanno?	1. Baambu waa<br>2. Tuubbote waa<br>3. Huxxaminoha balete waa<br>4. Huxxaminokkiha balete waa<br>5. Huxxinoonniha buete waa<br>6. Huxxaminokkiha burqanno waa<br>7. Xeenu waa<br>8. Daadanno waa (lagga/xashshuwa)<br>9. Garba /kooficho/ga’rinowaa		

		10. Hayilaandete waa		
203	Way afamanno dargi hiikkooti?	1.Qae'yara<br>2. Hoowete giddoydinni<br>3. Wole darga		
204	Waa dirri'ne hinganni yanna mageeshsha adhanno?	Daqiiqunni/xu'eessunni . . . .<br>998. dibadoomma		
205	Sa'uta lame lamala giddo way bae barra wo'ma keeshshino?	2. Eewa<br>0. Dee'ni<br>998. dibuuxoomma		
206	Way co'ichimma agarsiisate atewayinni agarrannire assoottori/tari no yite hedatta/o?	1.Eewa<br>0. Dee'ni<br>998. dibuuxooma		
207	Maatekki duucha yannara hiittooha shumate mine horoonsidhanno?<br>MITEEKKITE KULA FOKKITURO, DARGU GEESHSHA SA'E LAI	WAYINNI XAADINSOONNITA/ XAADINSOONNIKKITA<br>1. Baamba fanne horoonsi'nannita<br>2. Baambaho xaaddino way maashine<br>3. Ummooni balera waa horoonsi'ra<br>4. Base baalate waa horoonsi'ratenni<br>5. Shumate mini waa hiissine horoonsi'nanniro diafoommo/a<br>6. Baaldete waa<br>7. Gottiima shumate mine<br>8. Shumate mini dino/xawoho/ishinu giddo		
208	Konne shumate mine wole maateno horoonsidhanno?	1. Eewa<br>0. Dee'ni		
209	Maatekki ledo woloota maate me''e horoonsidhanno?	1. 10 nni ajjannori<br>2. 10 woy hakkuy ali<br>998. dibuuxoomma		
210	Shumate mine loonsoonnihu hiikkooti?	1. mini giddooti<br>2. hoowenke giddooti<br>3. wolewaati		

211	Maatekkira sagale loosi’rate hiittenne giirate wolqa horoonsidhinanni?	1.Korreente<br>2. Liquide gase<br>3. Kalaqamu gaaze<br>4.Obbi-kose<br>5.Laambunni<br>6.kasaletenni<br>7. haqqetenni<br>8. kasheetenni<br>9. mu’rote agadinni<br>10. saadate obbinni<br>11.mine sagale diloosi’neemmo		
212	Duucha wote sagale minenso, barandahonso woy wolu baxxino mine loosidhinanni?	1. mine<br>2. wole baxxino mine<br>3. barandaho<br>4. woleno, xawisi-------------------		
213	Baxxinohu sagale qishidhinanni mini noo’ne?	1. eewa<br>0. dinonke		
214	Galtinanni mini cira ayeho?	1.Aneho<br>2. Baante gallanniho<br>3. Maatenni<br>4. Fiixu/ elunniho<br>5. Woleno xawisi…………..		
215	Mini’nera maatekki goxate horoonsidhanno kifilla me’’e nooho?	-------------------------		
216	Minu giddoydo iimiidi raga (korniise) mayinni biifinsoonniho?	1. Kalaqamu haqqinni (biinfori dino, sabbunni,nna kandoonni sabbinni)<br>2. Calla anfi gede assinnoonniha (xu’minsoonnikki saattinni/plastic shara,shomboqqotenni/leemmichu nni, haqqu xaawulinni, and komborsaatunni)		

		3. Xu'minsoonni korniise (siwiilunni/culku siwiilinni, haqqunni, alumineemetnni/ simintotenni		
217	Mini uulliidi loosaminohu mayinniiti?	1. Kalaqamu haqqinni (biinfori dino, s<br>2. Calla anfi gede assinnoonniha (xu'n xaawulinni, and komborsaatunni)<br>3. Xu'minsoonni korniise (siwiilunni/c (minxaafe)		
219	Minu anni / ati saadate hoshsho, baatto hawuurranni saadanna lukkuwa ceo noosiho?	1.eewa<br>0. dinoe	222	
220	Aliidi saada giddonni meessi'nehu mageeshshaati?<br>Nookkiha ikkiro '00'.<br>95 nna woy roore ikkiro '95'.<br>Buunxoonnikkiha ikkiro '98'.<br>a. Ado saadanna bootta?<br>b. Wole saada?<br>c. Farado/harre/gaangootta?<br>d. Gaala/<br>e. Me''e?<br>f. Ge'reewo?<br>g. Caacurre/ lukkuwa?<br>h. Diishshote koshsha?	a. Ado saadanna bootta?<br>b. Wole saada?<br>c. Farado/harre/gaangootta?<br>d. Gaala/<br>e. Me''e?<br>f. Ge'reewo?<br>g. Caacurre/ lukkuwa?<br>h. Diishshotekoshsha?		
221	Saada galtannohu baxxino mini noo'ne?	1.eewa<br>0.dinoke		
222	Maatekkira meessi'neti/ki'neti loosidhinanni baatto noo'ne?	1.eewa<br>0. dinonke	224	
223	Maatekkira loosi'nanni baatto me''e hekitaare ikkitanno?	--------------------------Hekitaareeti		
224	Maatekkira:<br>1. Korreente?	Yes No		

	2. Raadoone?<br>3. Telewishiine?<br>4. Mine silke?<br>5. Kompiitere?<br>6. Mitore qiissanno udiinnichi?<br>7. Xarapheezzu?<br>8. Barcimu?<br>9. Shiwote daallasi?<br>10. Korreentete wolqanni sagale raisi'nanni mixashsho?<br>11. Laambunni/shaamu/? Faanoose	1. Korreente ……..1 0<br>2. Radoone……….1 0<br>3. Televishiine …….1 0<br>4. Mini bilbili……….1 0<br>5. kompiyuutere…….1 0<br>6. Motore qiissaancho...1 0<br>7. xarapheeza ………1 0<br>8. barcimu……………1 0<br>9. shiwote daallasi ….1 0<br>10. Siwiilu mixashsho…1 0<br>11. Laambu. Shaamu, faanoose lamp……………….1 0		
225	Maatekki mereero aanino udiinni noosari no:<br>a) Tolobishiine --------<br>b. Bilbilu------<br>c. Shalleette------------<br>d. Xexxerrisu------<br>e. Saadate gaare-<br>f. Kaameelu/ hogowi kaameeli -----<br>g. Yowolo motor nooti-----<br>h. Sasu goommi baajaaje ------------	Yes No<br>a) Televishiine . . . . . . 1 0<br>b) Mobayle …. . . . . . 1 0<br>c) Shalleette . . . . 1 0<br>d) Xexxerrisu. . ……….1 0<br>e) faradu/harrete gaare .1 0<br>f)kaameelu/hogowikaameeli..1 0<br>g) Yowolo motore nooti. . 1 0<br>h) sasu goommi baajaaje… 1 0		
226	Maatekkira baankete akkowaante kiiro noonsa?	1. Eewa<br>0. Dinonke		
227	Maatekki giddo shiimmaadda daddalu uurrinsha akkowaante kiiro noonsa?	1. Eewa<br>0. Dinonke		

Mojule III: Mashalaqqisaanonnita Sirote Gade

A.K	Xa’mubba	Dawarote Bebbeehache	sai/ kubbi	buuxo
301	Adhamitta yannara me’’e diro ikkannohe?	__________________________ 998. diqaagamannoe		
302	Umi godowi gatihe yannara me’’e diro ikkannohe?	__________________________ 998. diqaagamannoe		
303	Godowu gate egenninohe?	1. Eewa<br>0. Diegennoomma	3	
304	Aliidi xa’mote kiiro dawarokki ‘’Eewa ‘’ ikkituro, 303, me’’e higge?	---------------------------		
305	Umo ka’e egennootta?	0. Dee’ni<br>1. Eewa	307	
306	Aliidi xam’mote kiirora 305, dawarokki ‘Eewa’ ikkituro, me’’e higge?	----------------------------		
307	Ooso ilte egennootta?	0. Dee’ni<br>1. Eewa	311	
308	Aliidi xa’mote kiiro 306 dawarokki ‘’Eewa’ ikkituro, me’e higge?	-----------------------------		
309	Iloottari mereerinni lubbote noohu me’’eho?	------------------------------		
310	Minekki ilootta qaaqquulli kiiro me’’eho?	---------------------------		
311	Tenne mule gatihe godowi yannara musi’roottati /xisso xaaddinohe? (Law. shuma giirannonna gaangaanna darshiishanno dhibba)	0. Dee’ni<br>1. Eewa		
312	Maatekki mereero bikkiweelo mundeete xiiwo leeltinohu no?	0. Dino<br>1. Eewa no	3	
313	Aliidi xa’mo 312 dawarokki ‘’Eewa’’ ikkituro, isi ayeti?	2. Anna’yaati<br>3. Ama’yaati		

		4. Meya rodoo'yaati<br>5. Ahaahe'yaati<br>6. Woleno xawisi………………..		
314	Godowu giddo ree ilamino qaaqqi noohe (godowamihunni 28 lamala?	1. eewa<br>0. dinoe<br>998. diqaageemma		
315	Qaaqquullu ilantay heedhe reyte egentinohe (mitte lamala wo'minsakkinni)?	1. eewa<br>0. dee'ni<br>998. diqaageemma	gooffino	
316	Aliidi xa'mote kiiro 314 dawarokki ''eewa '' ikkitro, me''e hige xaadinohe?	_____________		
317	Aliidi xa'mote kiiro 314 dawarokki ''eewa '' ikkitro, qaaqqullu bushaawihehu dargu hiikkooti?	1. mineeti<br>2. fayyimmate uurrinsha giddooti<br>3. wolewano xawisi _________		

Mojule IV: Amuwu Keeraanchimma Agarate Owaante

A.K	Xa'mubba	Dawarote bebbeehache	sai/kubbi	Buuxo
401	Gedeno gatihe godowa mixo mixidheeti?	1. eewa<br>0. dee'ni	405	
402	Aliidi xa'mote kiiro 401 dawarokki 'deeni' ikkituro, kora ikkiheri maati?	1. yannitte ilate damboowishshi doogga horoonsi'ra hoogate<br>2.Horoonsi'roommana diloossinoe<br>3. Woleno xawisi …………………		
403	Aliidi xa'mote kiiro 401 dawarokki ledde 'dee'ni' ikkituro, takkonta dibaxooma?	1. eewa<br>0. dee'ni		
404	Aliidi xa'mote kiiro 401 dawarokki ledde 'dee'ni' ikkituro, aganu gedensa ila hasi'ratta?	1. eewa<br>0. dee'ni		
405	Gedeni qaaqqi godowi gatihe yannara xisso macciishshantuhe hige?	1. eewa<br>0. dee'ni	 407	
406	Aliidi xa'mote kiiro 405 dawarokki ''eewa'' ikkituro, maati xissokki?<br>(doorsha nabbabboti)	1. Akkimale siimunni du'nama<br>2. Bikku aliidi bisu iibbabbo<br>3. Siimu qooxeessi foole gana<br>4. Huxano<br>5. Damuume goola<br>6. Anga/ alba fuuga<br>7. Duucha yanna looqaleessa<br>8. Akkimale mundeete xiiwo<br>9. Woleno xawisi_________		
407	Gedeni qaaqqo godowitta yannara fayyimmate owaante afi'ritta?	1.eewa<br>0. dee'ni	 409	
408	Aliidi xa'mote kiiro	1. Godowu gatanno yannara xaaddanno		

	407dawarokki ‘eewa’ ikkituro, towaanyote koru maati?	mitiimma<br>2. Godowu ledo xaado nookki fayyimmate qarra<br>3. Ilate albiidi buuxo<br>4. woleno (xawisi) -----------------		
409	Gedeni qaaqqi godowi gatihe yannara ANC buuxo assi’rootta?	1. eewa<br>0. diaasi’roomma<br>998. diqaageemma	 425<br>425	
410	Aliidi xa’mote kiiro 409 dawarokki ‘eewa’ ikkituro, ANC buuxo me’’e higge assi’rootta?	____________<br>998.diqaagamaae	 412	
411	ANC buuxo assi’rootta yanna aanti-gedeno agadhite xawisi?	1mi ANC ……………….aganira<br>2ki ANC ………………aganira<br>3kkiANC………………..aganira<br>4ki ANC……………….aganira<br>999. diqaageema		
412	ANC owaante hiikkiinni afi’rattta?	1. mootimmate hospitaalenni<br>2. hallanyu hospitaalenni<br>3. Halaale irkidhe kaa’litanno wolqa<br>4. Fayyimmate mereershi<br>5. hallanyu xaggate mine<br>6. Halaale irkidhino xagga mine<br>7. Fayyimmate keella<br>8. woleno xawisi) ________________		
413	Aliidi xa’mote kiiro 412 owwante mayra doodhitta?	1. mini’yra mule ikkeenna<br>2. jawaata fayyimmate uurrinsha kalaqate<br>3. woleno xawisi _________		
414	Gedensi godowi gatihe yanna ANC buuxo assihehu ayeti?	1.Dottorra<br>2. Nerse		

		3. Ilshiishaano meenti<br>4. Fayyimmate biiro<br>5. fayyimmate<br>6. budu ilshiishaano<br>7. wole xawisi		
415	Gedeni qaaqqi godowi gatihe yanna Titaanosete kitiwate qasirota?	1. eewa<br>0. deee’ni<br>998. diqaageemma		
416	Aliidi xa’mote kiiro 415 dawarokki ‘eewa’ ikkituro, me’e higee?	---------------------------------------		
417	Gedeni qaaqqi godowi gatihe yanna Laaboraatorrete miirmmara assirotaa?	1. eewa<br>0. deee’ni<br>998. diqaageemma		
418	Aliidi xa’mote kiiro 415 dawarokki ‘eewa’ ikkituro, hiitee?	1= Mundee (Mundeete dana)<br>2= Mundeee (Shufuurrunita)<br>3= Jawa Shumma<br>4= Wayi Shumma<br>5= Wole xawisi		
419	Gedeni qaaqqi godowi gatihe yanna ANC buuxo assir’rootta?	1. eewa<br>0. deee’ni<br>998. diqaageemma	 425<br>425	
420	Aliidi xa’mote kiiro 419 dawarokki ‘eewa’ ikkituro, sokkate tajekkina?	1= Unuuna Qansa<br>2= Busha Dano Amuwunnita<br>3= Maatete Damboowishshi Mixo<br>4= Ilate Owaante<br>5= Qaaqqu ilamay hee’re busha malaatta leellisha<br>6= Qaaqqu ilamay hee’reenna keeraanchimmasi agara<br>8= Woleno Xawisi………………………	 421	

421	Hiittooha busha xissote malaate qummeessinihe?<br>(Doorsha nabbabbooti)	1 = kaajjado umu damuume<br>2 = Mitiri illete lame ikka<br>3 = Qaaqqu godowu giddo godo’la giwa<br>4 = bikkiweelo mundeete xiiwo<br>5 = Alba darsha<br>6 = Anganna alba darsha<br>7 = huxano<br>8 = Sirote bisi mundee xooho<br>9 = boode wodanu xisso<br>l0 = aliidiri giddo dino		
422	Aliidi xa’mote kiiro 421 te Busha xisso malaati mitiimma leeltuheta ha’rattara amaalihe?	1.eewa<br>0. dee’ni<br>998. diqaagamaae		
423	Gedeni qaaqqo hiikko ilattaro qummeessinoonnihe?	1. eewa<br>0.dee’ni<br>998. diqaageemma	 428<br>428	
424	Aliidita xa’mote kiiro 420 dawarokki ‘’eewa’ ikkituro, hiikko ilatta gede kulloonihe?	2. mine<br>3. fayyimmate keellira<br>4. fayyimmate mereershira<br>5. hallanyu xagga mine<br>6. meessi fajjo hakimewa<br>7. mootimmate hospitaalera<br>8. hallanyu hospitaalera<br>9. meessi fajjo hospitaalera<br>10. Woleno xawisi………………		
425	Ilitta wote ilshiishaahe ogeessa qeechinoonnihe?	1. eewa<br>0. dee’ni<br>998. diqaagamaae	 427<br>427	
426	Aliidi xa’mote kiiro 425, dawarokki ‘’eewa’ ikkituro, aye ilshiishahera qeechinihe?	1. Dottoru<br>2. Nerse//ilshiishaano/fayyimmate ogeeyye		

		3. Xeena ekistenshiine<br>4. Budu ilshiishaano<br>5. Woleno xawisi__________		
427	Ilate barri iilahera alba togoo qixxaawo assootta?	1. hodhishsha<br>2. miinja suuqi’rootta<br>3. mundee aannoheha<br>4. mittoreno		
428	ANC towaanyo assi’roottakiha ikkiro, korkaata kulattae?<br>(hassiisirodawarohalashshi)<br>(doorsha nabbabbooti)	1. ANC lainohunni egennote anjewoy hoonge<br>2. keeraanchimmate qarri xaada hooga<br>3. fayyimmate uurrinsha galeemma mninni fafa<br>4. horoonsiraanote batinyinni<br>5. fayyimmate ogeeyye egennote anje<br>6. hodhishshu qarrini<br>7. fayyimmate uurrinsha mareemmata yanna hoogatenni<br>8. woleno xawisi ---------------------		
429	Fayyimmate uurrinsha giddo ilate mixidhe egennootta?	1. eewa<br>0. deeni<br>998. diqaageema	431	
430	Aliidi xa’mote kiiro 429 te dawarokki ‘dee’ni’ ikkituro, korkaata kulattae?<br>(dawaro hasiissuro duucha assi)<br>(doorsha nabbabbooti)	1. Fayyimmate mine ilate daafira huwanyo hooga<br>2. fayyimma kisannori xaada hooga<br>3. fayyimmate uurrinsha mini’ya wiinni fa<br>4. fayyimmate ogeeyye egenno anje<br>5. hodhishshu qarra<br>6. woleno xawisi---------------------		
431	Gedeni qaaqqi ilami wote ateno ikki maatekki hiitte qixxaawo assitini?	1. hodhishshunnita<br>2. womaashsha suuqi’ra<br>3. keeraaanchimma buuxi’ra owaante		

		4. mundee aannoha qixxeessi'ra<br>5. dandoo noosi ogeessa qixxeessi'ra<br>6. mittoreno		
432	Gedeni qaaqqo hiikko ilitta?	1. Mine<br>2. Fayymmate keellira<br>3. Fayyimate mereershira<br>4. Hallanyu kilinikera<br>5. Meessi fajjo kilinikera<br>6. Mootimmate hospitaalera<br>7. Hallanyu hospitaalera<br>8. Meessi fajjo hospitaalera<br>9. Woleno xawisi ………………	442	
433	Gedeni ilakki fayyimate uurrinshra iloottaha ikkiro, mamootenna mamote ha'runsatta?	1. game hanaffo yannara<br>2. game hanaffuhunni 6-12 saate gedensaanni<br>3. game hanaffuhunni 13-18 saate gedensaanni<br>4. game hanaffuhunni 19-24 saate gedensaani<br>5. woleno xawisi __________		
434	Gedeni qaaqo ilita yaannara hiissiennahe ilitta?	1. Mitii'mummakki iloomma<br>2. udiinnichu kaa'lonni iloomma<br>3. Godowa darreennae iloomma<br>4. woleno xawisi __________		
435	Gedeni qaaqqo ilshiishihehu ayeti?	1.Dottoraho<br>2. Nersete<br>3. Ilshiishaanote<br>4. Dagate Fayyimmate ogeeyyeeti<br>5. Fayyimmate ekistenshineeti<br>6. Budu ilshiishaanooti (TBA)<br>7. Woleno xawisi___________		

436	Gedeni qaaqqo ilitta yannara mulekki ikke kaa’lamihehu noohe?	1. Eewa<br>0. dee’ni		
437	Aliita xa’mote kiiro 433te dawarokki ‘’eewa’’ ikkituro, ayeti ilitta yannara ka’lamihehu?<br><br>Wolurino hee’riro?<br><br>WO’MANTA TUMO HINKIILI	1. ama’ya<br>2. ballo’ya<br>3. meyya rodoo’ya<br>4. beetti’ya beetto<br>5. wolootu balluwi’ya<br>6. wolootu annuwi<br>7. wolu fiixi firi<br>8. Neighbour<br>9. Woleno xawii……………………		
438	Ilitta yannara aante noo assootubba assinoonnihe?	1. mundee aa<br>2. shota agatto<br>3. marfe<br>4. sirote biso dare halashsha<br>5. Ilate game rakkanno gede assanno xagicho		
439	Marfe qasi’roottaro, maahooti? (doorsha nabbabbooti)<br><br>BAALANTA TUMO HINKIILI	1= game ajishate<br>2 = mundee uurrisate<br>3= huxano uurrissate<br>4= woleno xawisi)<br>999= dibadooma		
440	Ilate yannara ga’manni mageeshsha keeshshannohe?	1. 12 saate woro<br>2. 12- 24 saate<br>3. 25- 36 saate<br>4. 37 – 48 saate<br>5. 48 saate ale		
441	Gedeno ilami qaaqqi hiittoohu ilami?	1. lubbote noohu<br>2. bushaawinohu		

		3. fayyu ilame darganko reyno		
442	Aleenni xa’mote kiiro 429 te, qaaqqo mine iloottaro, mine ila mayra doodhitta?<br>(doorsha nabbabbooti)	1. boode yanna calla ga’minoehura<br>2. mule fayyimmate owaante nookkihura<br>3. hodhishshu mitiimma noo daafiraati<br>4. Hodhishshaho womaashshu hooginoehuraati<br>5. Fayyimmate ogeeyye laafa ikkitinohuraati<br>6. muli fiixi’yanna jaalla’ya nooehuraati<br>7. Owaante uynanni gara waajjeetii (Sirote bisi kifile halashsha lawinore)<br>8. fayyimmate uurrinsha fojo maaxawokkihuraati<br>9. fayyimmate uynanni owaante horo afa hoogeeti<br>10. Wole manni olluunnihu amaaleennaeeti<br>11. woleno xawisi _____________		
443	Gedini ila game yannara wole xisso xaaddinoheti no?	1. ee<br>0. dino<br>999. diqaagamaae	456	
444	Aliidi xa’mote kiiro 440te dawarokki ‘’ee’’ ikkituro, may xaadinohe?<br>(Do not read the choices)	1 = akkimale sirote bisi mundee<br>2 = busha foole gana<br>3 = akkimale bisu iibbabbo<br>4 = qaaqqu balaxe anga woy lekka abba<br>5= Baby bad position/ mal presentation<br>6= seeda yanna game (> l2 saate)<br>7= hobbaate keeshsha<br>8 = Otoottote gurdama<br>9= Suuwo gobba higge fula<br>10 = Suuwo goowaho xaaxama		

		11= huxano<br>l2 = Bisu heleelamme xaada<br>l3= doorshu giddo dino	456	
445	Hatte mitiimmara furgao woy hurre hasidhe egennootta?<br>Buuxo: hiikkonne mine heedhe assi’rootta gargarooshshe	1. Eewa<br>0. Dee’ni	456	
446	Aliidi xa’mote kiiro 442te dawarokki ‘’eewa’ ikkituro, xagga hiikkiinni hasi’rootta)?<br>AMA XAGGA HASIDHINOHA IKKIRO, MITTE DAWARO CALLA QOLI	1.Mine<br>2. Fayymmate keellira<br>3. Fayyimate mereershira<br>4. Hallanyu kilinikera<br>5. Meessi fajjo kilinikera<br>6. Mootimmate hospitaalera<br>7. Hallanyu hospitaalera<br>8. Meessi fajjo hospitaalera<br>1. Woleno xawisi ………………		
447	Aleenni xa’mote kiiro 442te dawarokki ‘’eewa’’ikkituro, xagisihehu ayeti?<br>MITTE DAWARO CALLA HINKIILI	1. Dottoru<br>2. Nerse<br>3. Ilshiishaano<br>4. Fayyimmate ogeeyye<br>5. Fayyimmate extenshine<br>6. Budu ilshiishaano (TBA)<br>7. Maate<br>8. Elunna emu<br>9. Qachu manni<br>10. Woleno xawisi___________		
448	Maroottawa mitiimma xaaddeennahe wole hospitaale qolle sonkoonnihe?<br>MINEETIRONNA GOBBAATIRO QORQORI	1.Eewa<br>0. Dee’ni	455	

449	Aleenni xa'mote kiiro 448 te, hiikka sonkihe?	1. Fayyimmate keella<br>2. Fayyimmate mereersha<br>3. Hospitaale<br>4. Magano huuccitaariwa<br>5. Woleno xawisi……………………..		
450	Wolewa sonkiheha korkaata kultinohe?	1. Eewa<br>0. Dee'ni	451	
451	Aliidita xa'mote kiiro 447te dawarokki ''eewa'' ikkituro, mayra wolewa sokkuheyya?<br>BAALANTA TUMO HINKIILI	1 = Dare gowate udiinni hoonginni<br>2 = Bikkiweelo mundeete xiiwo<br>3 = Woyyaabbino buuxo assi'rate<br>4 = Dottorra hooggeenna<br>5 = Mundee tareessa<br>6 = Qaaqqu godowaho ofolla gari<br>7 = Mitu qaaqquulli kalaqami<br>8 = Qaaqqu cili amate otoottora<br>9 = Otoottote birxe fa'nama hooga<br>10 = Wolere xawisi<br>999 = Dafoomma		
452	Hakkonne darga marootta?	1. Eewa<br>0. Dee'ni<br>999. diqaageemma	457	
453	Aliidi xa'mora dawarokki eewaancho ikkituro, mageeshshi yanna agadhite gargadhatta?	________________		
454	Marte egennoottakki darga woshshante marittaro, macciishshammoheri no?	1. Eewa<br>0. Dee'ni<br>999. diqaagamannoe		
455	Sonkoonnihe darga ha'ra cee'mattahu?	1 = Meentu busha dancha bada hoogatenni<br>2 = Minaanni huwata hoogatenni<br>3 = Owaantete dargi xeertinyinni<br>4 = Hodhishshu qarrinni		

		5 = Qaaqqu fayyimma hoonge<br>6= Bikka sa'no womaashsha baantannihura<br>7 = Hanqata owaante<br>8 = Hiikka mara noohero afootto<br>9 = Ha'rate yanna dinoe<br>10 = Womaashshu hoonginni<br>1 l = Woleno xawisi<br>999 = Diafoomma		
456	Godowinni nootta yannara hodhishshaho doorsha assi'ratta?	1. Eewa<br>0. Dee'ni		
457	Suuqqi'rootta womaashsha horoonsidhe egennootta?	1. Eewa<br>0. Dee'ni		
458	Konne qaaqqo ilittahunni 6 ki lamala geeshsha xaadduhe mitiimma?	1. eewa<br>0. dee'ni<br>999. diqaagamannoe	460	
459	Aliidi xa'mote kiiro 458 te dawarokki ''eewa'' ikkituro, maati xaaadiheri?<br>(doorsha nabbabbooti)	1 = Akkimale mundee otoottote giddonni xooha<br>2 = Busha foole gana<br>3 = Bikka sa'ino bisu iibbabbo<br>4 = Qaaqqu unuunni giddollicho maaxama<br>5 = 'Tetaanuse'<br>6 = Hobbaate keeshsha<br>7 = Kaajjado godowu xisso<br>8 = Huxano<br>9 = Gobbagino unuuna<br>10 = dawaro dino		
460	42 ki barra fayyimmate mini owaante afi'ratta?	1. eewa<br>0. dee'ni<br>999. I don't remember		
461	457 ki xa'mora rakkino owaante	1 ______ saate gedensaanni		

	afi'rattahu mamooteeti?	2. ______ barrubba gedensaanni<br>3. ______ lamala gedensaanni		
462	Aliidita xa'mote kiiro 457te dawarokki ''eewa'' ikkituro, 42 barri giddo ilate gedeni owaante me''e higge hasi'ratta	____________<br>998.Don't know/remember	412	
463	Umi yannara buuxo mama assi'ratta?	1. Mine<br>2. Fayyimmate Keellira<br>3. Fayyimmate mereershira<br>4. Hospitaalete<br>5. Woleno xawisi………………		
464	Wole lexxote owaante noohe?	1. Qaaqqoho kittiwaate aa<br>2. Maatete kiiro bikkaasincho mixo<br>3. Unuuna qansinanni gara amaala<br>4. woleno xawisi ____________		
465	Ilate gedeni owaante hiikkiinni afi'ratta?	1.Fayymmate keellira<br>2. Fayyimate mereershira<br>3. Hallanyu kilinikera<br>4. Meessi fajjo kilinikera<br>5. Mootimmate hospitaalera<br>6. Hallanyu hospitaalera<br>7. Meessi fajjo hospitaalera<br>8. Woleno xawisi ………………		
466	Gedeni qaaqqira ilami wote ilate gedeni owaante uyhehu ayeti?	1.Dottoraho<br>2. Nersete<br>3. Ilshiishaanote<br>4. Fayyimmate ogeeyyeeti<br>5. Fayyimmate ekistenshineeti<br>6. Budu ilshiishaanooti (TBA)<br>7. Woleno xawisi___________		
467	Galtekki hedo/ lao amate	1. Eewaanchote		

	fayyimmate owaante hiittoote?	2. Hi'naanchote<br>3. Diafoomma		
468	Minikki maate yannitte fayyimmate mini owaante ayee la'annote ?	1. Lamenka<br>2. Gashshaanna'ya<br>3. Anella<br>4. wole xawisi _____________		
461	Minikkira mule doogote injo noohe?	1.Eewa<br>0. Dinoe		
670	Qarichimmate qajeelsha maatekki adhitino?	1. Eewa<br>0. Dee'ni		

Mojule V: Amuwu Fayyimma Owaante Lainohunni Egennonna Laooshshe Xiinxallo Assinoonni Qooxeessira

1. Amuwu Fayyimma La'nohunni Assinanni Egennote Xiinxaawo

A.K	Xa'mubba	Dawarote bebbeehache	sai/kubbi	buuxo
	**Ilate balaxote buuxo lainohunni egenno buuxa**			
501	Ilate balaxote buuxo owaante maatiro afootta?	0. Dee'ni<br>1.Eewa	503	
502	Aleenni xa'mote kiiro 501te dawarokki ''eewa'' ikkituro, ilate balaxote buuxo amuwu otootto keeraanchimmara horo noose?	0. Dee'ni<br>1. Eewa		
503	Umi doycho ilate balaxote buuxo towaanyo xaphi yitannohu mamooteeti?	1.16ki lamala sa'eenna<br>2. 16ki lamala wo'mikki		
504	Xaphooma ilate balaxote buuxo towaanyo meyati godobbe ilta geeshsha noota afootta?	1.4nni ajjanno<br>2. 4 nna hakkuy ale		
505	Umiti woyyaabbino ilate balaxote buuxo towaanyo assi'roottahu hiikkooti?	2. Fayyimmate keellira<br>3. Fayyimmate mereershira/hospitaalete		

506	Ilate balaxote towaanyo yannara godowii noo ama labaratorete buuxo assidhanno?	0. Dee’ni<br>1. Eewa		
507	Ilate balaxote towaanyo yannara godowii noo amara xagga hasiissanno?	0. Dee’ni<br>1. Eewa		
508	Ilate balaxote towaanyo yannara godowii noo amara amaalete kaa’lo hasiissanno?	0. Dee’ni<br>1. Eewa		
	**Fayyimmate Uurrinsha Giddo Ilate Mayimma Lainohunni Shiqqino Xa’mubba**			
509	Fayyimmate uurrinshara ilate hornyi maatiro afootta?	0.diafoomma<br>1. eewa		
510	Allidita xa’mote kiiro 501te dawarokki ‘’eewa’ ikkituro, uurrinshate gidoo ila amuwahonna ilantanno qaaqquullira hornyaamete?	0. Dee’ni<br>1. Eewa		
511	Hiitte manchooti Fayyimma uurrinsha ilatta kultinoheti?	1. Danotenni<br>2. Baalu		
512	Ilate seyanno dargi hiikkooti?	1.Mineeti<br>2. Fayyimmate uurrinsha		
513	Qaaqquulle ilate injo qineessora ayeti dandoo noosihu?	1.Anfoonnikki manni (TBA), elunna emu , jaalto/jaala<br>2. Fayyimmate mini ogeeyye		
514	Ilate yannara xaaddinose xissonni mannu aalo ikkitino ama no?	0. Dino<br>1. Eewa		
515	Qajeeltino fayyimmate ogeeyye amuwu rewo gargartanno yite hedatta?	0. Dee’ni<br>1. Eewa		
	**Ilate gedeni towaanyo Lainohunni Egenno (Huwanyo) Badate Xa’mubba**			
516	Ilate gedeni towaanyo daafira afoottari no?	0. Dino		

		1. Eewa		
517	Aliidi xa’mote kiiro 501te , dawarokki ‘’eewa’’ ikkituro, Ilate gedeni amuwunna qaaqquullu keeraanchimmara horaameettete	0. Dee’ni<br>1. Eewa		
518	Ilate gedeni towaanyo me’’e hinge assi’nanni agarranniro afootta?	1. 3nni ajjanno<br>2. 3 nna hakkuy ale		
519	Umi yannara ilate gedeni towaanyo assate tolanno dargi hiikkooti?	2. Fayyimmate keellaati<br>3. Fayyimmate mereersha / hospitaalete		
520	Godowii noo ama PNC towaanyo yannara wole lexxote amaale hasidhanno?	0.Dee’ni<br>1. Eewa		
521	Ilate gedeni yannara xaaddanno mitiimmanni ama reytanno yite hedatta?	0. Dee’ni<br>1. Eewa		
522	Ilate gedeni towaanyo assi’ra amuwu rewo gargartanno yite hedatta?	0. Dee’ni<br>1. Eewa		

2. Amuwu Fayyimmate Owaante Daafira Beeqqaanote Noonsa Lao Badate Shiqqino Xa’muwa

1-takkonta sumuu yaa giwa; 2- sumuu yaa giwa 3- mereerima; 4- sumuu yaa ;

5- Addanko sumuu yaa

A.K	Xa’mubba	1	2	3	4	5
	**Laote xa’mubba Ilate balaxote lainohunni**					
523	Ilate balaxote towaanyo amuwahonna qaaqqu suuwora horaameessa ikkasira mageeshsha sumuu yaatta?					
524	Umi doycho Ilate balaxote towaanyo 16 lamalanni ajjannoha ikkiro, mageeshsha sumuu yaatta?					
525	4 calla hige Ilate balaxote towanyo assi’ra gatino godowira ikkitanno; yitanno hedora mageeshsha sumuu yaatta?					
526	Umi doycho Ilate balaxote towaanyo assi’rate injaanno dargi fayyimmate mereershaati/ hospitaalete yitanno hedora					

	mageeshsha sumuu yaatta					
527	Ilate balaxote towaanyo yannara godowinni noo amuwi labaratorete buuxo assi’ra noonsata mageesshsha sumuu yaatta?					
528	Ilate balaxote towaanyo yannara godowinni noo amuwi wole hasiissanno xagga qixxeessinansara hasi’rannota mageeshsha sumuu yaatta?					
529	Ilate balaxote towaanyo yannara godowinni noo amuwi wole hasiissanno amaale uynansara hasi’rannota mageeshsha sumuu yaatta?					
530	Amuwu fayyimmate owaantera Ilate balaxote towaanyo assi’rannokkihu, womaashshu fulo batidhanno yee hedayti yitanno hedo mageeshsha irkisatta?					
531	Amuwu fayyimmate owaantera Ilate balaxote towaanyo assi’rannokkihu, fayyimmate mini ogeeyye mishshannonke yee hedayti yitanno hedo mageeshsha irkisatta?					
	**Laote Xa’mubba Fayyimmate Uurrinsha Giddo Ila Lainohunni**					
532	Amuwahono ikko ilamawo qaaqqira addinta jawa horo noose					
533	Amuwu fayyimmate uurrinshara ila danchate yite mageeshsha hedatta?					
534	Fayymmate uurrinshara qaaqquulle danchate yite hedatta?					
535	Fayyimmate ogeeyye qajeeltinota ikkansa mageeshsha sumuu yaatta?					
536	Ilate yannara babbaxxitino mitiimma rewoteno iillisha dandiitanno?					
537	Qajeeltino fayyimmate ogeeyyenni qaaqquulle ilshiisha amuwu rewo gargartanno?					
538	Ganto godowi keeraanchimma agarate mixo fushshi’ra					

	hasiissanno?					
539	Labbaahu fayyimmate ogeeyyenni ila saalaho woy seerimalete yite hedatto?					
540	Ilshiinshanni daallasira miteekkite game gadadissuro, disaallanni?					
541	Amuwu fayyimmate mine ila giwannohu, womaashshu fulo batidhanno yee hedayti yitanno hedo mageeshsha irkisatta?					
542	Fayyimmate ogeeyye amuwa ilshiishshanno yanna ayirrinye diuytanno yinanni hedo mageeshsha sumuu yaatta?					
	**Laote Xa’mubba Ilate Gedeni Towaanyo Daafira**					
543	Ilate gedeni towaanyo amuwahonna daaimaho danchate ikkase mageeshsha sumuu yaatta?					
544	3 calla hige Ilate gedeni towanyo assi’ra ilate yannara xaaddanno mitiimma hunate ikkitanno; yitanno hedora mageeshsha sumuu yaatta?					
545	Umi doycho Ilate gedeni owaanyo assi’rate injaanno dargi fayyimmate mereershaati/ hospitaalete yitanno hedora mageeshsha sumuu yaatta					
546	Godowii noo amuwira Ilate gedeni owaanyo assinanninsa yannara baxxitino amaale hasiissannonsa?					
547	Ilate gedeni towaanyora xaaddannose mitiimmanni ama reytannota mageeshsha sumuu yaatta?					
548	Ilate gedeni towaanyo fayyimmate uurrinshara amuwu rewo gargartanno yite mageeshsha hedatta?					
549	Amuwu fayyimmate owaantera PNC towaanyo assi’rannokkihu, womaashshu fulo batidhanno yee hedayti yitanno hedo mageeshsha irkisatta?					
550	Amuwu fayyimmate owaantera PNC towaanyo assi’rannokkihu, fayyimmate mini ogeeyye mishshannonke yee hedayti yitanno hedo mageeshsha irkisatta?					

Mojule VI: Dagoomi-budu Himano

A.K	Xa’mubba	Tumi-qolote bebbeehache	sai/kubbi	Buuxo
601	Qarqari’nera nooha dagoomi-bude Amuwu fayyimmate owaante heekki assinori maati?<br><br>(doorsha nabbabbooti)<br><br>BAALANTA TUMI-QOLO HINKIILI	1. Budu xagissaano hee’ranna iillishanno hekko<br>2.Amma’note xagissaano hee’ranna iillishanno hekko<br>3.Budunni ilshiishanno manni qooxeessaho hee’ra attendants<br>4.Maassamino way<br>5. Amu fayyimmate owaante horoonsha waajja<br>6.Waaxishshu hekko<br>7. MHSU lainohunni qooxeessu mdagoomira galtanno huwanyo<br>8. Gashshaanni iillishanno hekko<br>9. Balluwu ayiddi hekko<br>10. Woleno xawisi ………….		

Modele VII: Ilaannaho Uynanni Fayyimmate Owaante Horo Isilanchimara Noonsa Egenno Keennanni Borri-Xa'mo

<u>Ilate Balaxote Owaataasinu Harunso Silanchimma Daafira Shiqqino Borri-xa'mo</u>

1-lowo geeshsha diduushshanno.; 2- diduushshanno 3- mittoreno yaa didandeemmo; 4- danchaho 5- lowo geeshsha danchaho.

	**Xa'mo**	1	2	3	4	5
1	Fayyimmate ogeeyye ledo noohe miiloomikki hiittooho, ilate balaxote harunsokki yannara?					
2	Fayyimmate ogeeyye ledo noohe miiloomi guutinokkiha ikkiro, hiittoo ogeessaati yite hedatta?					
3	Fayyimmate ogeeyye ganyite uytanno owaante hasi'ratta woyte illacha tuge loosanno					
4	Fayyimmate ogeeyye, ilate balaxote harunso yannara ikkado yanna, uytanno yaatta towaanyokki yannara fayyimmate urrinshara ate hedora?					
5	Fayyimmate ogeeyye owaante uytanno yannara teeda agarate gade ilate balaxote owaante uynanni kifilera ate hedora hittooho yaate?					
6	Fayyimmate ogeeyyera hedokki xawisate noonsa gade ate hedora hittoote?					
7	Ate hedo gumulora beeqqootta ikkiro hiittoonni?					
8	Ilate balaxote harunsokki yannara, owaante afi'rate towaanyo assate agadhinanni yanna dikeeshshinanni yite hedatta?					
9	Fayyimmate uurrinsha wo'mankawa co'ittete ate hedora?					
10	Aterano daaimahono uynoonni fayyimmate owaataasinenni afi'rootta kaa'lo hiittoote? Lawishshu gede ilate yannara atenna daaimu co'ima agaratenni?					
11	Ilate balaxote harunsora uynanni yannara fayyimmate taje					

	ikkadote yite hedatta?					
12	Fayyimmate ogeeyyewinni ikkado taje uynanni ilate yine hendanni yannana godowu gatinkunni me’’e agana ikkiro, ikkadote yite hedatta?					
13	Ikkado taje afi’roomma yite hedatta, godowaho busha malaatta bade afate widoonni?					
14	Ate hedonni fayyimmate ogeeyye lifixxinote fayyimmate qara tirate?					
15	Ate hedonni ikkado tajenna xawishsha afi’roomma yite hedatta wo’munku fayyimmate qarrira?					
16	Ate hedora, ilate balaxote harunso assi’nanni kifile co’itete?					
17	Ate hedora, taaltino sagale saga’late ikkado taje afi’rooma yite hedatta?					
18	Ate hedora, TT (titanesete dhiba gargaranno kitiwaati) daafira ikkado taje noohe?					
19	Ate hedora, mundeete dani buuxo daafira ikkado taje nooe yite hedatta?					
20	Ate hedora, shumate buuxo daafira ikkado taje nooe yite hedatta?					
21	Ate hedora, kiilogiraamekkinna seendillikki bikko daafira ikkado taje noohe?					
22	Ate hedora mundeekki xiiwo bikkora ikkado taje nooe yite hedatta?					
23	Ate hedora, ikkadu xawishshi noohe uynannihe dinyera?					

Konni Woroonni Noo Borri-xa'mo Ama Ikkadonna Silancho Owaante Illtu Yannara Afi'rase Keennanni Xa'mo

1-lowo geeshsha sumuu diyeemma 2- sumuu diyeemma; 3- mittoreno diyeemma; 4- sumuu yeemma 5- lowo geeshsha sumuu yeemma

A.K	XA'MUWA	1	2	3	4	5
1	Mule'ne noo fayyimmate uurrinshara owaante afi'nara agadhinanni baychi danchaho yee hedeemmo					
2	Amuuwu kifilera, no shummate bale co'itete yee heedeemma mule noo'ne fayyimmate uurrinshara					
3	Mule no fayyimmate urinshara, anganna biso hayishi'nanni dargi no.					
4	Mule no fayyimmate uurrinshara kuriuu labbannori no, korreentenna way.					
5	Amuwu kifile e'nanni waalchinni hananfe mittore qarramikkinni/shotuniititi.					
6	Mule no uurrinshara, fayyimmate ogeeyye uytanno gametenna ilate yanna kaa'lo danchate.					
7	Mule no uurrinshara, fayyimmate ogeeyye uytanno daaimu/marqu keeranchimma daafira danchate.					
8	Mule no uurinshara, fayyimmate ogeeyye uyitanno kaa'lo xissote yannara danchate.					
9	Mule no fayyimmate uurinsha mageeshshi yanna adhanno lekkatenni?	________sa'ate				
10	Hodhishshu owaante afidhinanni fayyimmate uurinsha hadhinanni woyte.	1. Ee<br>0. Dee'ni				
11	Mule no fayyimmate uurrinshara, owaante afi'rate baantanni.	1. lowo batooshshe<br>2. mereerima<br>3. shiima				

Konni Woroonni Noo Borri-xa’mo Keennannihu Silancho Owaante Ilate Gedensaanni Noo Yannara Fayyimmate Uurrinshshara

1-lowo geeshsha diduushshanno; 2- diduushshanno; 3- mittorenno diyeemma; 4- danchaho 5- lowo geeshsha danchaho

	Xa’muuwa	1	2	3	4	5
1	Fayyimmate ogeeyye ledo noohe miiloomikki hittooho, ilate balaaxxote harunsokki yannara?					
2	Fayyimmate ogeeyye ledo noohe miiloomi guutinokkiha ikkiro, hittoo ogeessaati yite hedatta?					
3	Fayyimmate ogeeyye ganyite uytanno owaante hasi’ratta woyte illacha tuge loosanno.					
4	Ate hedora, xisso gargarate uyinanni xagichi no, ilate gedensiidi yannara.					
5	Fayyimmate ogeeyye owaante uytanno yannara teeda agaratenna ayirrisate gade ilate gedensaanni uyinanni owaante kifilera , ate hedora hittooho yaate					
6	Ate hedora,fayyimmate ogeeyye ledo hedokki xawisate hasaawatta					
7	Ate hedora,fayyimmate ogeeyye maatekkira leellishshino albi danchaho					
8	Ate hedora, qaaqqu ilame ka’’anni hee’reenna unuunna qansate orte hiittoote ilanni kifilera.					
9	Ate hedora, fayyimmate ogeeyye uytino owaante daaimu keraanchimmara ka’litanno.					
10	Ate hedora, afirootta owaante fayyimmate ogeeyyewinni fayyimmakkira lowo kaa’lo afidhino lawishshu gede ilate gedensaanni uynanni bisikki co’imara.					
11	Ate hedora, ikkado taje nooe yite hedatta qaaqqu					

	keeraanchimmara/fayyimmara.					
12	Ate hedora ikkado taje nooe yite hedatta ragunni unuuna qansate hayyora?					
13	Busha ilate widoonni dagganno malaatta bade afate daafira ikkado taje nooe yite hedatta ilate gedensiidi yannara, amaate keraanchimmaranna qaaqqu fayyimmara.					
14	Ate hedora, fayyimmate ogeeyye uytanno owaante lifixxinote qaqqu fayyimmara.					
15	Ate keraanchimara uynanni owaantera fayyimmate ogeeyyenni fayyimmte qarra bade afatenna tirate widoonni.					
16	Aye hajorano ikkado xawishshi nooe yite hedatta.					
17	Ate hedora fayyimmate mini co’imma lainohunni.					
18	Ate hedora shumate mininna hayishshi’nanni kifilla co’imma daafira.					
19	Ate hedora, ilate gedensaanni owaante afi’nanni baychi ikkadoho.					
20	Ate hedora owaataassinete ikkadu baychi no, xiwamaassinete ledo xaadinohunni.					
21	Ate hedora, ikkadu daallasi no gamete/ilanni kifilera.					
22	Ate hedora, ikkado ogeeyye noo kaa’litanohe gede.					
23	Ate hedora ilate gedensaanni afi’nanni woyte ikkado baychi no fooliishshi’rate mittu qarri nookkiha (caabbichu, hurote. kifilete loonsannirichinni).					

Mojule IIX: Ilate Albiidi yanna, Illanni yannaranna ilate gedensaanni xaadanno qarri daafira keenanni borri-xa’mo.

A.K	Xa’muwa	Dawaro	Sai	qaagiis hsha
801	Godowinni hee’neenna fayyimmate ogeeyyewiinni kaa’lo hasi’rano qarri maatiro ballo kulattae?<br><br>Wolunna?<br><br>(Mittunni aleenni doora dandinanni.)	1= deerra sa’ino umu damuume.<br>2= Mitiri illete la’nanni woyte duuccha ikka.<br>3=qaaqqu godowu giddo milli yaa hooga/aja<br>4= mundeete xiiwo lexxa<br>5= alba darsha.<br>6= albanna lekka darsha.<br>7= huxi’ra.<br>8= otoottote widoonni mundaa.<br>9= mudukku woroonni deerra aleenni xissa.<br>l0=aleeni noori doorshi giddo dino.		
802	Xaa kayinni kulattaeri, illanni yannara fayyimmate ogeeyyewiinni xagisi’ra hasi’ranno qarri maatiro ballo kulattae?<br><br>Woluna?<br><br>(Mittunni aleenni doora dandiinanni)	1 =Deerra sa’ino otoottote mundaa.<br>2 = Otoottotenni busha foole afi’rinori fula.<br>3 = Deerra sa’ino bisu iibbilli.<br>4 = Ilamanno qaaqqi anga woy lekkate widoonni daa.<br>5=Ilamano qaaqqi maqqaalame/ragiweelo daa.<br>6. Illanni yannara 12 saate aleenni godowu game/gamaa’ma.<br>7= Keere tidhama hooga.<br>8 = Otoottote gurdaama.<br>9= Suuwo, qaaqqo balaxxe		

		otoottotewinni fula.<br>10=Suuwo qaaqqu goowira xaaxante fula.<br>11= Bisu huxi’ranno yannara.<br>l2 = bisu heleelama.<br>l3= dawaro dino.		
803	Qaaqqo ama ilte ka’anni hedheeenna fayyimmate ogeeyyewiinni xagisi’ra hasiissanno qarri hiikkonneeti?<br>Woluna?<br><br>(Mittunni aleenni doora dandiinanni)	1 = Derra sa’ino otoottote mundaa.<br>2 =Otoottotenni bushu foole afi’rinori fula.<br>3 = Deerra saino bisu iibbilli.<br>4 = amate ununnu qacce giddo hige ea .<br>5 = Tetaanese<br>6 = Keere tidhama hooga<br>7 = mudukku woroonni deerra aleenni xissa.<br>8 = Bisu huxi’ranno yannara<br>9 = Amate unuuni darsha.<br>10 = dawaro dino		

Mojule IX: Ilatenna Ille Ka'ni Gedensaanni Daanno Qarri Balaxote Qixxaawote Rosicho

A.K	Xa'mo	Dawaro	Sai	qaagiishsha
901	Godobbe heedhe ikko ilattara albaanni ati woy maatekki mixidhinoonnihu hiikko mine ilateeti?	1= Ee<br>2= Dee'ni<br>999= Diqaageemma.	906	
902	Ee ikkiro xa'mo 901, hiittenne mixo mixidhinoonni ati woy maatekki?<br>(Doorshsha nabbabooti!<br>Wolu no?<br>Dawaro qoqqowi	l= Ilate mule no fayyimmate uurrinsha doodhoomma<br>2= Qajeeltino fayyimmate ogeeyyenni ileemma yee mixiroomma<br>3= womaashsha suuqiroomma , sorranniere qixxeess'iroomma<br>4=ikkannoha hodhishshu dana doodhooma<br>5=Mundee'ya dani ay ledo xaadannoro afi're noomma aannoeha		
903	Ilatta uurrinsha, hiikkiicho doodhita mixokkira?<br>Nabbabbooti doorsha!	1. Mine<br>2. Fayyimmate keellira<br>3. Fayyimmate agarooshshira<br>4. Hosipitaalete<br>5. Wole xawisi………………		
904	Ogeeyye doodhoottaha ikkiro ay iillishihe?<br>Nabbabbooti doorsha!	1. Dottoru<br>2. Nerse/ilshiishaano ogeeyye<br>3. Xeena ekistenshine<br>4. Budu ogeeyye<br>5. Wole xawisi_______________		
905	Hodhishshaho dodhootta ikkiro, ati doodhootta hodhishshinni haratta godowinni heedhe?	1. Ee<br>0. Dee'ni		
906	Womaashsha suqqirootta ikkiro, godowinni hedhe horonsi'roota?	1. Ee<br>0. Dee'ni		

Ha’runsokkira galaxxeemmo!
