# Supplementary material for: Community-Based Health Education Led by Women’s Groups Significantly Improved Maternal Health Service Utilization in Southern Ethiopia: A Cluster Randomized Controlled Trial
Source: Healthcare (Basel). 2024 May 18;12(10):1045. doi: 10.3390/healthcare12101045 (PMC11121210; doi:10.3390/healthcare12101045)
Supplement: Supplementary file 1 [file healthcare-12-01045-s001.zip › File S3.pdf]

Table S1: Description of maternal health care service use among women of reproductive age in the northern zone of Sidama region, Ethiopia, 2023 (N = 1,070)

Variables	Intervention group	Control group	Total	P- value
	N (%)	N (%)	N (%)	
At least one ANC				0.001
Utilized	489 (90.6)	355 (67.0)	844 (78.9)	
Non-utilized	51 (9.4)	175 (33.0)	226 (21.1)	
Number of ANC visits				0.001
0	51 (9.4)	175 (33.0)	226 (21.1)	
1-4	264 (48.9)	221 (41.7)	485 (45.3)	
5-7	21 (3.9)	18 (3.4)	39 (3.6)	
≥8	204 (37.8)	116 (21.9)	320 (29.9)	
Type of health facility visited during ANC follow up				
Government hospital	204 (37.8)	75 (14.2)	279 (26.1)	0.001
Health Centre	397 (73.5)	349 (65.8)	621 (58.0)	0.006
Reasons to prefer the above-stated health facilities				
Close to my house	401 (74.3)	251 (47.4)	652 (60.9)	0.001
Competent health worker	193 (35.7)	185 (34.9)	378 (35.3)	0.775
Plan to give birth in HF				
No	104 (19.3)	229 (43.2)	333 (31.1)	0.001
Yes	436 (80.7)	301 (56.8)	737 (68.9)	

Place of delivery				0.001
Home	84 (15.6)	203 (38.3)	287 (26.8)	
Health facility	456 (84.4)	327 (61.7)	783 (73.2)	
Mode of delivery				0.927
SVD	454 (84.1)	445 (84.0)	899 (84.0)	
Instrumental	62 (11.5)	59 (11.1)	121 (11.3)	
C/S	24 (4.4)	26 (4.9)	50 (4.7)	
Type of providers attended during childbirth				
Doctor	96 (17.8)	40 (7.5)	136 (12.7)	0.001
Nurse	145 (26.9)	66 (12.5)	211 (19.7)	0.001
Midwife	395 (73.1)	324 (61.1)	719 (67.2)	0.001
Health officer	23 (4.3)	17 (3.2)	40 (3.7)	0.365
TBA	66 (12.2)	145 (27.4)	211 (19.7)	0.001
At least one PNC				0.001
Utilized	353 (65.4)	276 (52.1)	629 (58.8)	
Non-utilized	187 (34.6)	254 (47.9)	441 (41.2)	
Number of PNC visits				0.001
0	353 (65.4)	276 (52.1)	629 (58.8)	
1-3	239 (44.3)	195 (36.8)	434 (40.6)	
≥4	114 (21.1)	81 (15.3)	195 (18.2)	
Other services during PNC use				
Child vaccination	239 (67.7)	167 (60.5)	406 (64.5)	0.061

Family planning	316 (89.5)	220 (79.7)	536 (85.2)	0.001
Counseling about breast feeding	283 (80.2)	178 (64.5)	461 (73.3)	0.001

Table S2: Multilevel modified Poison regression analysis result of a random intercept model for maternal health service use variation at cluster level in north zone of Sidama region, Ethiopia, 2023 (N = 1,070)

Measure of variation	Model 1 (95% CI)	Model 2 (95% CI)	Model 3 (95% CI)	Model 4 (95% CI)
**ANC related information**				
Variance of intercept	0.01 (0.001, 0.11)	0.002 (0.001, 1257.5)	0.009 (0.001, 0.005)	0.005 (0.001, 0.002)
ICC percentage	22.35 (12.38-36.97)			
Model fitness				
Log-likelihood ratio	-1043.49	-1028.08	-1033.27	-1019.96
AIC	2090.99	2092.17	2088.54	2083.92
BIC	2100.94	2181.73	2108.39	2093.38
**Health facility delivery related information**				
Variance of intercept	0.01 (0.01, 0.05)	0.02 (0.008, 0.961)	0.009 (0.008, 0.017)	0.001 (0.0001, 0.004)
ICC percentage	21.88 (11.40, 37.87)			
Model fitness				
Log-likelihood ratio	-1026.57	-1009.04	-1016.46	-1001.38
AIC	2057.14	2054.08	2054.93	2046.76

BIC	2067.09	2143.64	2074.78	2056.22
**Postnatal care related information**				
Variance of intercept	0.03 (0.006, 0.108)	0.021 (0.03, 0.113)	0.02 (0.001, 0.115)	0.01 (0.001, 0.134)
ICC percentage	10.76 (5.26, 20.75)			
Model fitness				
Log-likelihood ratio	-961.16	-933.85	-958.22	-932.21
AIC	1926.32	1931.71	1928.45	1918.43
BIC	1936.27	2028.14	1958.30	1932.76

ICC: Intra-class correlation coefficient; AIC: Akaike information criteria; BIC: Bayesian information criteria; CI: confidence interval.

Effect modification result of ANC

We entered the interaction terms in the final model for women's occupation and intervention status, husband occupation and intervention status, mass media use and intervention status, wealth index and intervention status, model family training and intervention status, place of residence and intervention status, cluster-level mass media use and intervention status, and cluster-level poverty and intervention status to see if women's occupation, husband occupation, mass media use, wealth index, model family training, place of residence, cluster-level mass media use, and cluster-level poverty modifies the effect of intervention. It is implied that there was no significant effect modification because none of the interaction terms were statistically significant.

Effect modification result of HFD

We entered the interaction terms in the final model for women's occupation and intervention status, husband occupation and intervention status, mass media use and intervention status, wealth index and intervention status, model family training and intervention status, place of residence and intervention status, cluster-level mass media use and intervention status, and cluster-level

poverty and intervention status to see if women's occupation, husband occupation, mass media use, wealth index, model family training, place of residence, cluster-level mass media use, and cluster-level poverty modifies the effect of intervention. None of the interaction terms was statistically significant, implying the absence of a significant effect modification.
